# De novo transcriptome and phytochemical analyses reveal differentially expressed genes and characteristic secondary metabolites in the original oolong tea (*Camellia sinensis)* cultivar ‘Tieguanyin’ compared with cultivar ‘Benshan’

**DOI:** 10.1186/s12864-019-5643-z

**Published:** 2019-04-03

**Authors:** Yuqiong Guo, Chen Zhu, Shanshan Zhao, Shuting Zhang, Wenjian Wang, Haifeng Fu, Xiaozhen Li, Chengzhe Zhou, Lan Chen, Yuling Lin, Zhongxiong Lai

**Affiliations:** 1 College of Horticulture, Fujian Agriculture and Forestry University, Fuzhou, 350002 China; 2 Institute of Horticultural Biotechnology, Fujian Agriculture and Forestry University, Fuzhou, 350002 China; 3 Anxi Tea Research Institute, Anxi, 362400 China

**Keywords:** *Camellia sinensis*, Transcriptome, Phytochemical, Secondary metabolites

## Abstract

**Background:**

The two original plants of the oolong tea cultivar (‘Tieguanyin’) are “Wei shuo” ‘Tieguanyin’—TGY (Wei) and “Wang shuo” ‘Tieguanyin’—TGY (Wang). Another cultivar, ‘Benshan’ (BS), is similar to TGY in its aroma, taste, and genetic make-up, but it lacks the “Yin Rhyme” flavor. We aimed to identify differences in biochemical characteristics and gene expression among these tea plants.

**Results:**

The results of spectrophotometric, high performance liquid chromatography (HPLC), and gas chromatography-mass spectrometry (GC-MS) analyses revealed that TGY (Wei) and TGY (Wang) had deeper purple-colored leaves and higher contents of anthocyanin, catechins, caffeine, and limonene compared with BS. Analyses of transcriptome data revealed 12,420 differentially expressed genes (DEGs) among the cultivars. According to a Kyoto Encyclopedia of Genes and Genomes (KEGG) analysis, the flavonoid, caffeine, and limonene metabolic pathways were highly enriched. The transcript levels of the genes involved in these three metabolic pathways were not significantly different between TGY (Wei) and TGY (Wang), except for two unigenes encoding IMPDH and SAMS, which are involved in caffeine metabolism. The comparison of TGY *vs*. BS revealed eight up-regulated genes (*PAL*, *C4H*, *CHS*, *F3’H*, *F3H*, *DFR*, *ANS*, and *ANR*) and two down-regulated genes (*FLS* and *CCR*) in flavonoid metabolism, four up-regulated genes (*AMPD*, *IMPDH*, *SAMS*, and *5′*-*Nase*) and one down-regulated *XDH* gene in caffeine metabolism; and two down-regulated genes (*ALDH* and *HIBADH*) in limonene degradation. In addition, the expression levels of the transcription factor (TF) PAP1 were significantly higher in TGY than in BS. Therefore, high accumulation of flavonoids, caffeine, and limonene metabolites and the expression patterns of their related genes in TGY might be beneficial for the formation of the “Yin Rhyme” flavor.

**Conclusions:**

Transcriptomic, HPLC, and GC-MS analyses of TGY (Wei), TGY (Wang), and BS indicated that the expression levels of genes related to secondary metabolism and high contents of catechins, anthocyanin, caffeine, and limonene may contribute to the formation of the “Yin Rhyme” flavor in TGY. These findings provide new insights into the relationship between the accumulation of secondary metabolites and sensory quality, and the molecular mechanisms underlying the formation of the unique flavor “Yin Rhyme” in TGY.

**Electronic supplementary material:**

The online version of this article (10.1186/s12864-019-5643-z) contains supplementary material, which is available to authorized users.

## Background

*Camellia sinensis* (L.) is an important economic crop which originated from the southwest of China. Its product, tea, is one of the three major non-alcoholic beverages worldwide [1]. Fujian Province (southeast China) is one of the most important tea production and marketing areas in China, and numerous national and provincial improved tea cultivars have contributed to the importance of tea in the local economy. *C. sinensis* cv. ‘Tieguanyin’ (TGY) originated from Xiping town, Anxi county, Fujian Province, China, and was identified as a Chinese national improved cultivar in 1985. The characteristic compounds of tea are catechins, caffeine, theanine, and volatiles, and these secondary metabolites are also important indicators of tea quality [2]. The TGY cultivar is the highest grade of oolong tea due to its unique “Yin Rhyme” flavor, which can be described as an elegant, gentle floral flavor, mellow and thick taste, and a sweet aftertaste. There are some reports that specific secondary metabolites may be related to the formation of the “Yin Rhyme” flavor in TGY tea [3, 4]. In the core area in which TGY originated, two of the earliest original tea plants of TGY are preserved; “Wei shuo” ‘Tieguanyin’—TGY (Wei) and “Wang shuo” ‘Tieguanyin’—TGY (Wang). These two original TGY tea plants are very similar in their physiological characteristics, but the differences in their biochemical characteristics and secondary metabolites are not yet clear. Another oolong tea national improved cultivar, *C. sinensis* cv. ‘Benshan’ (BS), also originated from Xiping town, Anxi county, Fujian Province, China. The aroma and taste characteristics of BS tea are very similar to those of TGY tea, with a strong aroma, endurable fragrance, and mellow, thick, and brisk taste, but it lacks the “Yin Rhyme” flavor. “Yin Rhyme” is the typical aroma and taste characteristics of TGY cultivar. The TGY and BS cultivars have similar phenotypes and quality characteristics, and have a close genetic relationship [5, 6]. These two tea cultivars and their tea products are sometimes confused. To date, there have been no reports revealing the major differences in metabolites between TGY and BS. In addition, the factors contributing to the formation of the “Yin Rhyme” flavor in TGY are not fully understood.

Transcriptome sequencing using next-generation sequencing technologies is a fast and cost-effective approach to generate genome-scale sequence resources [7, 8]. Consequently, this method is being used to analyze an increasing number of plant species and cultivars. Illumina sequencing technology, sequencing by synthesis, achieves high sequencing coverage at a lower cost, and has been used extensively for de novo transcriptome studies [9, 10]. High-throughput sequencing analyses have revealed many genes related to the flavor and quality of tea [11–13]. An RNA-seq study to identify differentially expressed genes (DEGs) among several tea cultivars revealed that many DEGs were involved in secondary metabolic pathways [12]. Tai et al. reported that DEGs between tea and oil-tea plants were related to major secondary metabolites (catechins, caffeine, and theanine), especially key genes at branch points in these three metabolic pathways, which might explain the differences in the contents of these three metabolites between tea and oil-tea. To date, however, little is known about the differences in secondary metabolites and DEGs between TGY and BS, and their related molecular mechanisms.

To reveal the varietal characters and quality characteristics of TGY (Wei), TGY (Wang), and BS, we used the two original tea plants of TGY and the original tea plant of BS as materials, and used custom RNA-seq to investigate DEGs between TGY and BS. We also analyzed a set of physiological indicators (anthocyanin, total carotenoids, catechins, caffeine, theanine, and limonene contents). These analyses provide important insights into the molecular mechanisms underlying secondary metabolite biosynthesis in TGY (Wei), TGY (Wang), and BS, and describe the phytochemical characteristics of the main metabolites. We also analyzed the relationship between differentially accumulated metabolites and their related DEGs between TGY and BS, and explored the formation of the “Yin Rhyme” flavor in TGY.

## Methods

### Plant materials and sample preparation

The original tea plants of TGY (Wei) (*C. sinensis* cv. ‘Tieguanyin’), TGY (Wang) (*C. sinensis* cv. ‘Tieguanyin’), and BS (*C. sinensis* cv. ‘Benshan’) used in this study were grown in Xiping town, Anxi county, Fujian province, China (Additional file 1: Figure S1). The first new sprouting fresh shoots and two leaves were picked from each plant and mixed separately. The tissues were frozen in liquid nitrogen immediately and kept in polyethylene bags at − 80 °C until further experiments.

### Investigation of main biological characteristics

The methods used to investigate the main biological characteristics of the three tea samples followed the national agriculture industry standard and are described in the following technical papers: “Technical code for evaluating crop germplasm tea plant (*Camellia sinensis*)” (NY/T 1312–2007) and “Guidelines for the conduct of tests for distinctness, uniformity and stability-tea (*Camellia sinensis*)” (NY/T 2422–2013).

### Determination of chlorophylls, carotenoids, and anthocyanin contents

Chlorophyll (Chl) and total carotenoids (Car) were extracted from tea leaves using acetone solution. Equal weights (0.1 g) of fresh leaves collected from three tea samples were immersed in 45% acetone (acetone: ethanol: distilled water = 4.5: 4.5: 1, 10 mL) solution for 12 h in the dark. After centrifugation at 10,000 g for 10 min, the absorbance of the extracts was read at 440 nm, 645 nm, and 663 nm using a spectrophotometer. Each sample was analyzed in triplicate. The Chl and Car contents were calculated according to the following formulae described by Deng [14]:

$$ \mathrm{Chl}\ \mathrm{a}\ \left(\mathrm{mg}/\mathrm{g}\right)=\left[\left(9.78{4\mathrm{OD}}_{663}-0.99{0\mathrm{OD}}_{645}\right)\ast \mathrm{V}\right]/\left({\mathrm{m}}^{\ast }1000\right) $$

$$ \mathrm{Chl}\ \mathrm{b}\ \left(\mathrm{mg}/\mathrm{g}\right)=\left[\left(21.42{6\mathrm{OD}}_{645}-4.65{0\mathrm{OD}}_{663}\right)\ast \mathrm{V}\right]/\left({\mathrm{m}}^{\ast }1000\right) $$

$$ \mathrm{Total}\ \mathrm{Chl}\ \left(\mathrm{mg}/\mathrm{g}\right)=\left[\left(5.13{4\mathrm{OD}}_{663}+20.43{6\mathrm{OD}}_{645}\right)\ast \mathrm{V}\right]/\left({\mathrm{m}}^{\ast }1000\right) $$

$$ \mathrm{Car}\ \left(\mathrm{mg}/\mathrm{g}\right)=\left[4.69{5\mathrm{OD}}_{440}-0.268\left(\mathrm{Chl}\ \mathrm{a}+\mathrm{Chl}\ \mathrm{b}\right)\ast \mathrm{V}\right]/\left({\mathrm{m}}^{\ast }1000\right) $$

Where V is the volume of mixed extraction solution (mL) and m is the sample mass (g).

Anthocyanins were also extracted from the three samples according to the method described by Zhou [15] with minor modifications. Briefly, 0.1 g fresh leaf tissue was ground into a powder in liquid nitrogen, and then extracted with 1 mL 0.1 mol/L hydrochloric acid in a water bath at 32 °C for 4 h. After centrifugation at 6000 g for 10 min, the supernatant was filtered through a 0.22 μm organic membrane, and absorbance was measured at 530 nm, 620 nm, and 650 nm against a blank of 0.1 mol/L hydrochloric acid solution. Each sample was analyzed in triplicate. The anthocyanin content was calculated according to the following formulae:

$$ {\mathrm{OD}}_{\uplambda}=\left({\mathrm{OD}}_{530}-{\mathrm{OD}}_{620}\right)-0.{1}^{\ast}\left({\mathrm{OD}}_{650}-{\mathrm{OD}}_{620}\right) $$

$$ \mathrm{Anthocyanin}\ \left(\mathrm{mg}/\mathrm{g}\right)=\left[{\mathrm{OD}}_{\uplambda}/\left(4.{62}^{\ast }{10}^4\right)\right]\ast \left(\mathrm{V}/\mathrm{m}\right)\ast {\mathrm{M}}^{\ast }1000 $$

Where OD_λ_ is the absorbance value of the anthocyanin extract after correction at 530 nm; V is the volume of mixed extraction solution (mL); m is the sample mass (g); and M is molar mass of anthocyanin (g/mol).

### Determination of total catechins, caffeine, theanine, and volatiles contents

Catechins, caffeine, and theanine were extracted and determined from three samples according to the method described by Tai et al. [16].

Catechins and caffeine were analyzed using a Waters 2695 high performance liquid chromatography (HPLC) system equipped with a 2489 ultraviolet (UV)-visible detector, and eluted catechins and caffeine were detected at 278 nm. The column temperature was 25 °C. Authentic standards of catechins and caffeine were purchased from Solarbio (Beijing, China). Each sample was analyzed in triplicate.

Theanine was analyzed using the same HPLC system equipped with a 2489 UV-visible detector and a 2475 fluorescence detector. The detection wavelength was set to 199 nm [17]. The column temperature was 25 °C. Authentic standards of theanine were purchased from Solarbio (Beijing, China). Each sample was analyzed in triplicate.

Volatiles were extracted from tea leaves and analyzed as described by Hu et al. [18]. A Clarus SQ 8 gas chromatograph-mass spectrometer (GC-MS; PerkinElmer, New York, NY, USA) fitted with an Elite-5MS capillary column (30 m × 0.25 mm × 0.25 μm) and a Turbmatix Headspace System (PerkinElmer) was used for analyses of volatile compounds. Data were analyzed using TurboMass 6.1 software (PerkinElmer). Each sample was analyzed in triplicate. Separated compounds were identified according to their retention indices and data in the NIST Mass Spectral Library and the Wiley Mass Spectral Library of Drugs, Pollutants, Pesticides, and Metabolites, using AMDIS Deconvolution software. The concentrations of volatiles in the tea cultivars were calculated as described by Hu et al. [18]. All data are expressed as mean ± standard deviation (SD).

### Total RNA extraction, library construction, and high-throughput sequencing

Total RNAs were separately extracted from TGY (Wei), TGY (Wang), and BS using a Trizol Reagent kit (Invitrogen/Life Technologies, Carlsbad, CA, USA) according to the manufacturer’s instructions. To identify the mRNAs in tea plants, a pooled RNA library was generated from the original tea plants (TGY (Wei), TGY (Wang), and BS). Extracted RNAs were quantified using a NanoDrop 2000 spectrophotometer (Thermo Scientific, Wilmington, DE, USA) and checked for integrity on an Agilent 2100 bioanalyzer (Agilent Technologies, Palo Alto, CA, USA) by denaturing agarose gel electrophoresis with ethidium bromide staining. The three libraries were constructed and sequenced by Biomarker Technologies (Beijing, China) using the Illumina HiSeq-2500 platform. Other details are as described by Lai et al. [19].

### De novo transcriptome assembly, functional annotation, and classification

To obtain clean reads from the three samples, the raw reads were first pre-screened to remove adapter sequences, reads containing more than 5% unknown nucleotides (N), and low-quality reads (those with > 20% of bases with a quality value of < 15) using SOAPnuke software (https://github.com/BGI-flexlab/SOAPnuke; parameters: -l 15; −q 0.2; −n 0.05). The trimmed and size-selected reads were then de novo assembled using the Trinity package (https://github.com/trinityrnaseq/trinityrnaseq; parameters: -SS_lib_type FR; −min contig length 200) [20]. According to the method described by Kim [21], hierarchical indexing for spliced alignment of transcripts 2 (Hisat2; https://github.com/infphilo/hisat2; parameters: -phred64; −sensitive; −no-discordant; −no-mixed; −I 1; −X 1000) was used to calculate the coverage of the three groups of tea transcriptome data relative to the two published versions of the tea genome [2, 22].

For gene annotation and protein prediction, all unigenes were searched against the Non-Redundant protein (NR), Swiss-prot, Gene Ontology (GO), Clusters of Orthologous Groups of proteins (COG), and Kyoto Encyclopedia of Genes and Genomes (KEGG) databases by BLAST (E-value < 1.0E^− 6^), The results of KEGG orthology analyses in the KEGG database of unigenes were obtained using KOBAS 2.0 [23]. Getorf [24] was used to find the coding DNA sequence (CDS) of each unigene longer than 200 bp. After predicting the amino acid sequence of each unigene, we used HMMER software [25] to compare it with the pfam database to obtain annotation information. We used HMMER software to identify transcription factor (TF) domains from the plant TF database (PlantTFDB), and TF family characteristics were identified according to the regulations described in the database (http://planttfdb.cbi.pku.edu.cn/) [26].

Bowtie software (https://github.com/BenLangmead/bowtie2; parameters: -q; −phred64; −sensitive; −dpad 0; −gbar 99,999,999; −mp 1,1; −np 1; −score-min L,0,-0.1; −I 1; −X 1000; −no-mixed; −no-discordant; −p 1; −k 200) [27] was used to align reads from each sample to the unigene library. Gene transcript levels were estimated using RSEM software (https://github.com/deweylab/RSEM; parameters: -default) [28]. The DEGs among the three samples were identified using EBSeq software (https://github.com/lengning/EBSeq) [29]. And *P*-values were adjusted using the FDR value [30]. The thresholds for judging the significance of differential gene expression were FDR ≤ 0.01 and |log_2_ (fold change)| ≥ 1. The DEGs were subjected to GO and KEGG enrichment analyses. For a given unigene, fragments per kilobase of exon per million fragments mapped (FPKM) values in the three tea plants were generated from each of the three transcriptomes. Major metabolic pathways in these three tea samples were identified by enrichment factor analyses. We classified DEGs according to KEGG classifications, and conducted enrichment analyses using clusterProfiler (https://github.com/GuangchuangYu/clusterProfiler; parameters: -gene; −*P*-value ≤0.01; −readable TRUE). Then, we calculated the FDR for each *P*-value. In general, the terms with an FDR < 0.01 were defined as significantly enriched. The enrichment results were visualized using ggplot2 (https://ggplot2.tidyverse.org/reference/geom_point.html).

### Identification of production of anthocyanin pigment 1 (PAP1) TF and phylogenetic tree construction

The amino acid sequence of the *Arabidopsis thaliana* PAP1 protein was downloaded from PlantTFDB [26], and used for BLAST searches of our transcriptome data. Local BLAST searches were conducted using BioEdit [31] with E-value < 1.0E^− 6^. We downloaded the Hidden Markov Model (HMM) file of the PAP1 domain (Pfam accession number: PF00249) from the Pfam database and used HMMER 3.0 software [25] with the default settings to perform an alignment search of PAP1 TFs. The results of these analyses were verified by local BLAST searches. We also confirmed the identity of CsPAP1 TF using tools at the InterPro database (http://www.ebi.ac.uk/interpro/).

To understand the structure of CsPAP1, the protein sequences of PAP1 from different plants were aligned by ClustalW with the default parameters. A phylogenetic tree was constructed using MEGA 7.0 with the neighbor-joining method with 1000 bootstrap replications [32]. Evolutionary distances were computed using the Poisson correction method. The function of the CsPAP1 TF was predicted using tools at the PlantTFDB.

### Relative expression analyses of selected genes

Total RNAs were reverse-transcribed using the PrimeScript RT reagent Kit (Takara, Otsu, Japan). To determine the relative transcript levels of the DEGs, the cDNA samples from the above-mentioned different tissues and samples under different treatments were diluted as the template, and then qRT-PCR analyses were conducted using gene-specific primers. The two-step qRT-PCR had a 20-μL reaction system consisting of 10 μL SYBR Premix Ex Taq II (TakaRa), 0.8 μL gene-specific primers, 1.0 μL cDNA, and ddH_2_O up to 20 μL. A Light Cycler 480 instrument (Roche, Switzerland) was used to detect gene transcripts. The qRT-PCR reactions included initial denaturation at 94 °C for 3 min followed by 40 cycles of denaturation at 94 °C for 10 s; annealing for 30 s, and extension at 72 °C for 15 s. Different annealing temperatures were used for different genes. Standard curves were obtained using a five-times dilution gradient of the mixed samples, and the reliability of the standard curve was determined. The *GAPDH* and *β-actin* genes were used as internal controls. Relative transcript levels were calculated using the 2^-ΔCt^ formula [33]. The primer sequences were designed using Primer 3 software (Additional file 2: Table S1). All qRT-PCR analyses were performed with three biological and technical replications.

### Statistical analyses

Each experiment represents three independent biological replicates, and all data are expressed as mean ± standard deviation (SD). Differences among groups were tested using one-way ANOVA and Duncan’s test, and significant differences among various groups are represented by different letters. Lowercase letters indicate significant difference (*P* < 0.05); uppercase letters indicate highly significant difference (*P* < 0.01). The data were analyzed using SPSS 20 software.

## Results

### Biological characteristics of tea cultivars

**Fig. 1:**
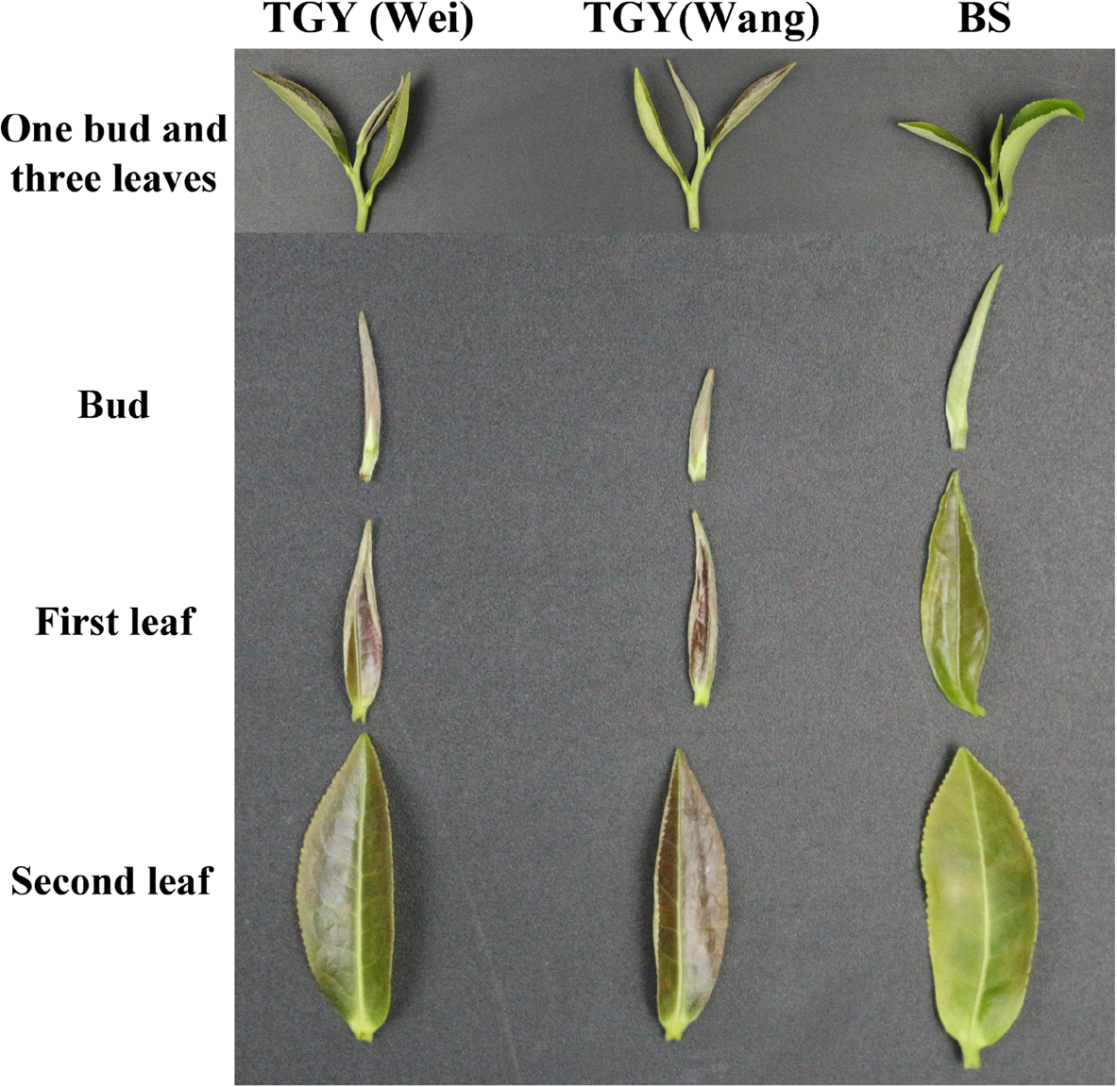
Leaf phenotype of “Wei shuo” ‘Tieguanyin’ (*Camellia sinensis* cv. ‘Tieguanyin’), “Wang shuo” ‘Tieguanyin’ (*C. sinensis* cv. ‘Tieguanyin’), and ‘Benshan’ (*C. sinensis* cv. ‘Benshan’). TGY (Wei): “Wei shuo” ‘Tieguanyin’ (*C. sinensis* cv. ‘Tieguanyin’); TGY (Wang): “Wang shuo” ‘Tieguanyin’ (*C. sinensis* cv. ‘Tieguanyin’); BS: ‘Benshan’ (*C. sinensis* cv. ‘Benshan’)

Both TGY (Wei) and TGY (Wang) are shrub-type, medium leaf, late-sprouting tea plants, but they showed some differences in their biological characteristics. The colors of the buds, first leaves, and second leaves of both TGY plants were green with purple (Fig. 1). The traits of leaf length, leaf width, and hundred-bud weight (one bud and three leaves) differed between TGY (Wei) and TGY (Wang). The leaf length and width were 7.96 cm and 3.09 cm, respectively, in TGY (Wei), compared with 7.77 cm and 2.92 cm, respectively, in TGY (Wang) (Additional file 3: Table S2). Therefore, the leaves of TGY (Wei) were longer and wider than those of TGY (Wei). The hundred-bud weight of TGY (Wang) was 165.0 g, which was 27.2 g heavier than that of TGY (Wei) (137.8 g).

The BS cultivar is a shrub-type, medium-leaf, medium-sprouting tea cultivar, which is very similar genetically to TGY [5, 6]. Compared with TGY, BS had earlier germinating and picking periods. The germinating and picking periods of TGY (Wei) and TGY (Wang) were similar, and differed by only 2 days. The bud color of BS was light green, unlike the purple bud of TGY (Wei) and TGY (Wang). The leaf color of BS was light green with lighter purple coloring than that of TGY (Wei) and TGY (Wang) leaves. The leaves of BS were shorter in length but wider than those of TGY (Wei) and TGY (Wang). Thus, the leaf shape of BS was different from the long oval shape of TGY leaves. The hundred-bud weight of BS was 98.2 g, lower than those of TGY (Wang) and TGY (Wei).

### Analysis of chlorophylls, carotenoids, and anthocyanin contents in ‘Tieguanyin’ (Wei), ‘Tieguanyin’ (Wang), and ‘Benshan’

**Fig. 2:**
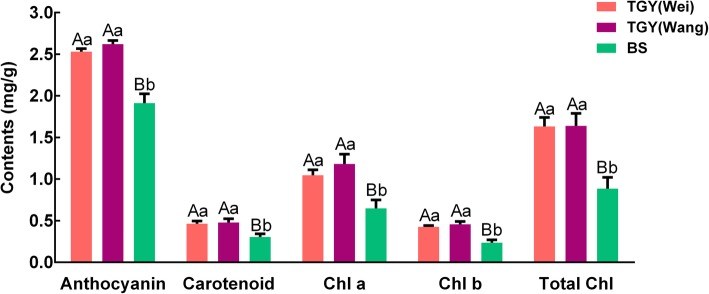
Contents of anthocyanin, carotenoids, chlorophyll a, chlorophyll b, and total chlorophylls in three tea samples. Chl a: chlorophyll a; Chl b: chlorophyll b; Total Chl: total chlorophylls. Data are means ± standard deviations (SD). Lowercase letter indicates significant difference (*P* < 0.05); uppercase letter indicates highly significant difference (*P* < 0.01)

To explore the reasons for the differences in leaf color among TGY (Wei), TGY (Wang), and BS, we determined the contents of major pigments, including chlorophylls, carotenoids, and anthocyanin. The anthocyanin contents in TGY (Wei), TGY (Wang), and BS were 2.53 mg/g, 2.63 mg/g, and 1.91 mg/g, respectively (Fig. 2). The anthocyanin contents were not significantly different between TGY (Wang) and TGY (Wei), but were significantly higher in TGY than in BS.

The total chlorophylls, chlorophyll a, and chlorophyll b contents were also higher in TGY (Wei) and TGY (Wang) than in BS. The carotenoids contents were significantly higher in TGY (Wei) (0.46 mg/g) and TGY (Wang) (0.47 mg/g) than in BS (0.30 mg/g), but were not significantly different between TGY (Wei) and TGY (Wang). These results confirmed that the contents of anthocyanin, carotenoids, and chlorophylls were higher in TGY than in BS.

### Total catechins, caffeine, theanine, and volatiles contents in ‘Tieguanyin’ (Wei), ‘Tieguanyin’ (Wang), and ‘Benshan’

Catechins, caffeine, and theanine play significant roles in the taste and flavor of tea, and the volatiles content is an important factor for determining tea aroma and quality [34, 35]. To explore the differences in these compounds among the three tea plants, the contents of catechins, caffeine, and theanine were determined by HPLC and the volatiles were analyzed by GC-MS.

**Fig. 3:**
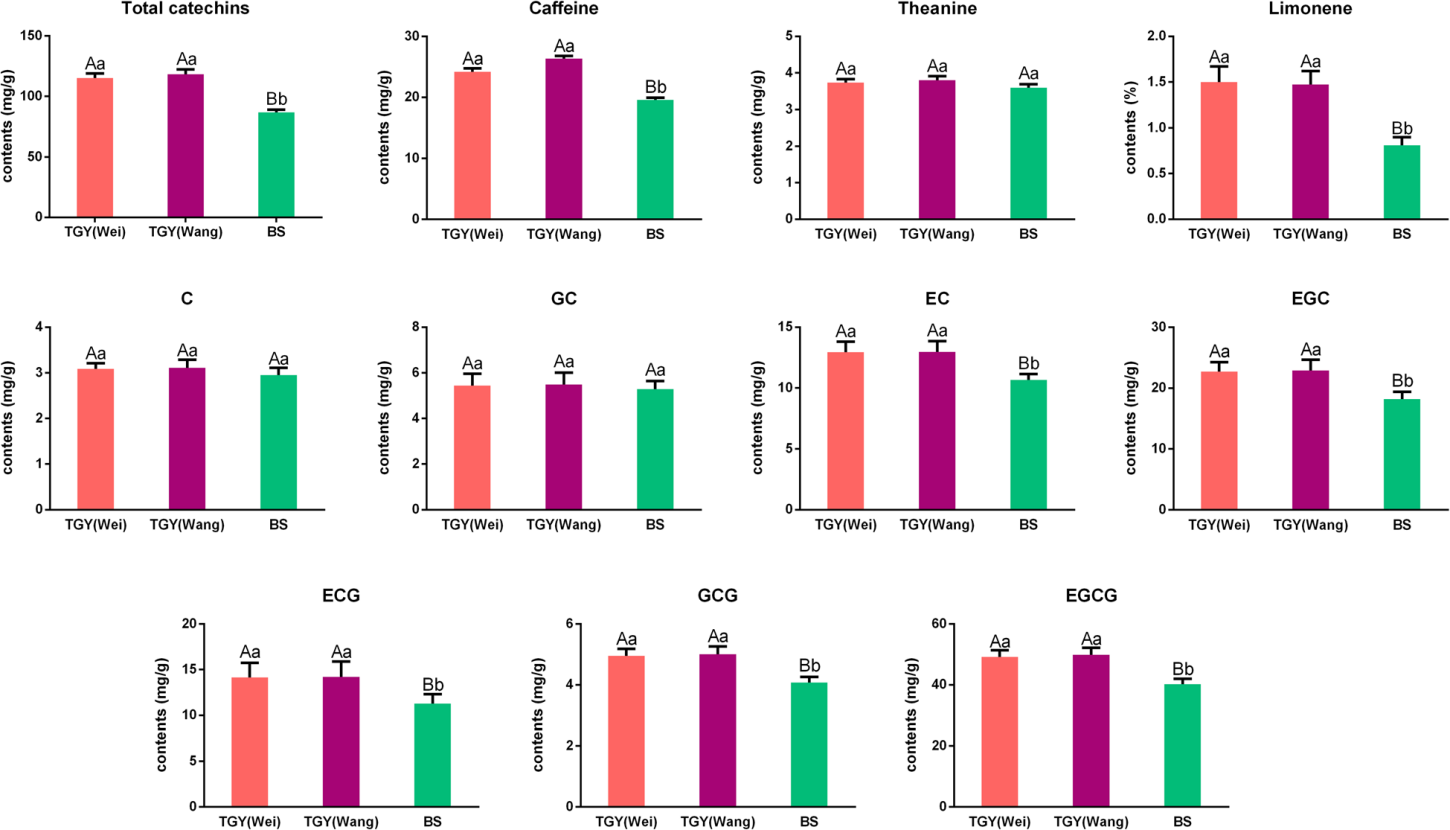
Contents of total catechins, caffeine, theanine, and limonene in three tea samples. Data are means ± standard deviations (SD). Lowercase letter indicates significant difference (*P* < 0.05); uppercase letter indicates highly significant difference (*P* < 0.01)

The total catechins contents were 115.21, 118.35, and 87.12 mg/g in TGY (Wei), TGY (Wang), and BS, respectively (Fig. 3). Among all the gallocatechin-type catechins, including gallocatechin (GC), epigallocatechin (EGC), gallocatechin gallate (GCG), and epigallocatechin gallate (EGCG), EGCG was the most abundant at concentrations of 49.12, 49.81, and 40.19 mg/g in TGY (Wei), TGY (Wang), and BS, respectively. The main catechin, EGCG, accounted for about 42% of total catechins in the three tea samples. Apart from catechin (C) and catechin gallate (CG), other catechins including epicatechin (EC), EGC, epicatechin gallate (ECG), GCG, and EGCG showed significantly higher concentrations in TGY (Wei) and TGY (Wang) than in BS. Overall, most catechins were present at higher concentrations in TGY (Wei) and TGY (Wang) than in BS.

The caffeine contents were significantly higher in TGY (Wei) (24.16 mg/g) and TGY (Wang) (26.36 mg/g) than in BS (19.56 mg/g), but did not differ significantly between TGY (Wei) and TGY (Wang). The theanine contents in TGY (Wei), TGY (Wang), and BS were 3.73, 3.80, and 3.59 mg/g, respectively. Statistical analyses showed that there was no significant difference in theanine contents between TGY (Wei) and TGY (Wang), between TGY (Wei) and BS, and between TGY (Wang) and BS. Thus, the difference in theanine accumulation among the three tea plants was very small.

The top 10 volatile components in TGY (Wei), TGY (Wang), and BS detected by GC-MS were verbenone, terpinolene, methyl cyclopentene, hexyl hexanoate, limonene, benzene acetaldehyde, alpha-farnesene, ocimene, methyl salicylate, and cyclohexadiene (Table 1). Except for limonene, there were no significant differences in the contents of the other nine volatile aroma compounds between TGY and BS. The limonene contents in TGY (Wei) and TGY (Wang) were 2.55 and 2.47%, respectively, significantly higher than that in BS (1.43%). The high concentrations of catechins, caffeine, and limonene may be critical factors affecting tea flavor and aroma characteristics, especially the unique “Yin Rhyme” flavor of TGY.

**Table 1:** Relative contents of top 10 volatile components in TGY and BS (%)

Volatile compounds	TGY (Wei)	TGY (Wang)	BS
Verbenone	19.59 ± 0.66^aA^	19.82 ± 0.87^aA^	19.03 ± 0.78^aA^
Terpinolene	12.49 ± 0.34^aA^	12.55 ± 0.37^aA^	12.56 ± 0.58^aA^
Methyl cyclopentene	10.45 ± 0.37^aA^	10.44 ± 0.44^aA^	10.40 ± 0.50^aA^
Hexyl hexanoate	5.80 ± 0.27^aA^	5.86 ± 0.32^aA^	5.58 ± 0.25^aA^
Limonene	1.50 ± 0.17^aA^	1.47 ± 0.15^aA^	0.81 ± 0.09^bB^
Benzene acetaldehyde	1.18 ± 0.10^aA^	1.20 ± 0.12^aA^	1.11 ± 0.10^aA^
Alpha-farnesene	0.85 ± 0.07^aA^	0.90 ± 0.08^aA^	0.83 ± 0.07^aA^
Ocimene	0.53 ± 0.04^aA^	0.55 ± 0.04^aA^	0.52 ± 0.04^aA^
Methyl salicylate	0.21 ± 0.02^aA^	0.22 ± 0.03^aA^	0.20 ± 0.02^aA^
Cyclohexadiene	0.18 ± 0.02^aA^	0.20 ± 0.02^aA^	0.19 ± 0.02^aA^

Lowercase letter indicates significant difference (*P* < 0.05); uppercase letter indicates highly significant difference (*P* < 0.01)

### Sequencing and de novo assembly of transcriptome data

After removing adapter sequences, low-quality reads, and reads with a high content of unknown base N (N% > 5%) from the raw data, a total of 15.03 Gb clean data were obtained by RNA-seq of the TGY (Wei), TGY (Wang), and BS libraries. We obtained 26,931,717, 21,533,093, and 26,690,143 clean reads from TGY (Wei), TGY (Wang), and BS (Table 2). For the TGY (Wei), TGY (Wang), and BS datasets, the Q30 percentages (sequencing correct rate ≥ 99.9%) were 88.30, 88.37, and 88.68%, respectively; and the GC percentages were 45.09, 45.21, and 45.13%, respectively. The coverages of TGY (Wei), TGY (Wang), and BS transcriptome data relative to the whole tea genome (*C. sinensis* var. *assamica*) [22] were 86.42, 86.42, and 85.99%, respectively; and relative to another tea genome (*C. sinensis* var. *sinensis*) [2] were 84.28, 84.35, and 82.97%, respectively. According to coverage criteria [21], more than 70% coverage is acceptable.

**Table 2:** Summary of sequencing reads after filtering

Samples	Read Number	Base Number	GC (%)	Q30 (%)
TGY (Wei)	26,931,717	5,385,659,400	45.09%	88.30%
TGY (Wang)	21,533,093	4,306,157,026	45.21%	88.37%
BS	26,690,143	5,337,098,059	45.13%	88.68%

All high-quality reads were assembled using the Trinity program. After assembly, 241,903 transcripts and 118,637 unigenes were obtained (Table 3). The N50 of transcripts and unigenes was 1640 and 1044, respectively. Of the assembled unigenes, the majority (64.28%) had lengths distributed between 250 and 500 bp. The length of 21,093 genes was > 1000 bp. We also obtained read count data for the transcriptome dataset (Additional file 4: Table S3). These values indicated that the sequencing data had sufficient quality for accurate assembly and adequate transcriptome coverage.

**Table 3:** Results of statistical analyses of transcriptome data

Length Range	Transcript	Unigene
200–300	59,445 (24.57%)	45,889 (38.68%)
300–500	47,799 (19.76%)	30,376 (25.60%)
500–1000	50,589 (20.91%)	21,279 (17.94%)
1000–2000	52,574 (21.73%)	14,516 (12.24%)
2000+	31,496 (13.02%)	6577 (5.54%)
Total Number	241,903	118,637
Total Length	236,530,814	76,976,903
N50 Length	1640	1044
Mean Length	977.79	648.84

### Functional annotation and classification of unigenes in the transcriptome

For gene function annotations and protein prediction, all unigenes were aligned to the non-redundant (NR), Swiss-Prot, Cluster of Orthologous Groups (COG), Gene Ontology (GO), and Kyoto Encyclopedia of Genes and Genomes (KEGG) databases using the BLAST program. Functional annotation analyses revealed that 16,330 (28.54%), 42,562 (74.39%), 2433 (21.73%), 36,422 (63.65%), and 56,939 (99.51%) unigenes had significant hits in the COG, GO, KEGG, Swiss-Prot, and NR databases, respectively.

In the COG function classification, unigenes were categorized into 25 clusters, The top five functions were “general function prediction only”, “replication, recombination and repair”, “transcription”, “signal transduction mechanisms”, and “posttranslational modification, protein turnover, chaperones” (Additional file 5: Figure S2). Based on GO classification analysis, 42,562 genes were annotated to three major categories: cellular component, molecular function, and biological process. The top three subgroups in the cellular component category were cell part, cell, and organelle. In the molecular function category, most unigenes were annotated into two subgroups: catalytic activity, and binding and transporter activity. In the biological process category, most unigenes were classified into three subgroups: metabolic process, cellular process, and single-organism process (Additional file 6: Figure S3).

In the functional annotation analysis, 50,077 CDS were detected by getorf prediction. Also, 1462 unigenes in 56 TF families were predicted. The five TF families with the largest number of genes were the basic helix-loop-helix (bHLH), ethylene-responsive factor (ERF), GRAS, C2H2, and MYB-related TF families, with 113, 104, 90, 88, and 84 genes, respectively (Additional file 7: Figure S4).

### Analysis of DEGs in TGY (Wei) *vs*. TGY (Wang), TGY (Wei) *vs*. BS, and TGY (Wang) *vs*. BS based on KEGG database

To identify DEGs among the three tea samples, we compared and analyzed the three sets of tea transcriptome data. In total, 12,420 DEGs were found. As mentioned above, most DEGs showed similar transcript levels in TGY (Wei) and TGY (Wang), but different transcript levels in BS compared with TGY (Wei) and TGY (Wang) (Additional file 8: Figure S5). To clarify the roles of these DEGs, enriched metabolic pathways were identified in TGY (Wei) *vs*. TGY (Wang), TGY (Wei) *vs*. BS, and TGY (Wang) *vs*. BS.

**Fig. 4:**
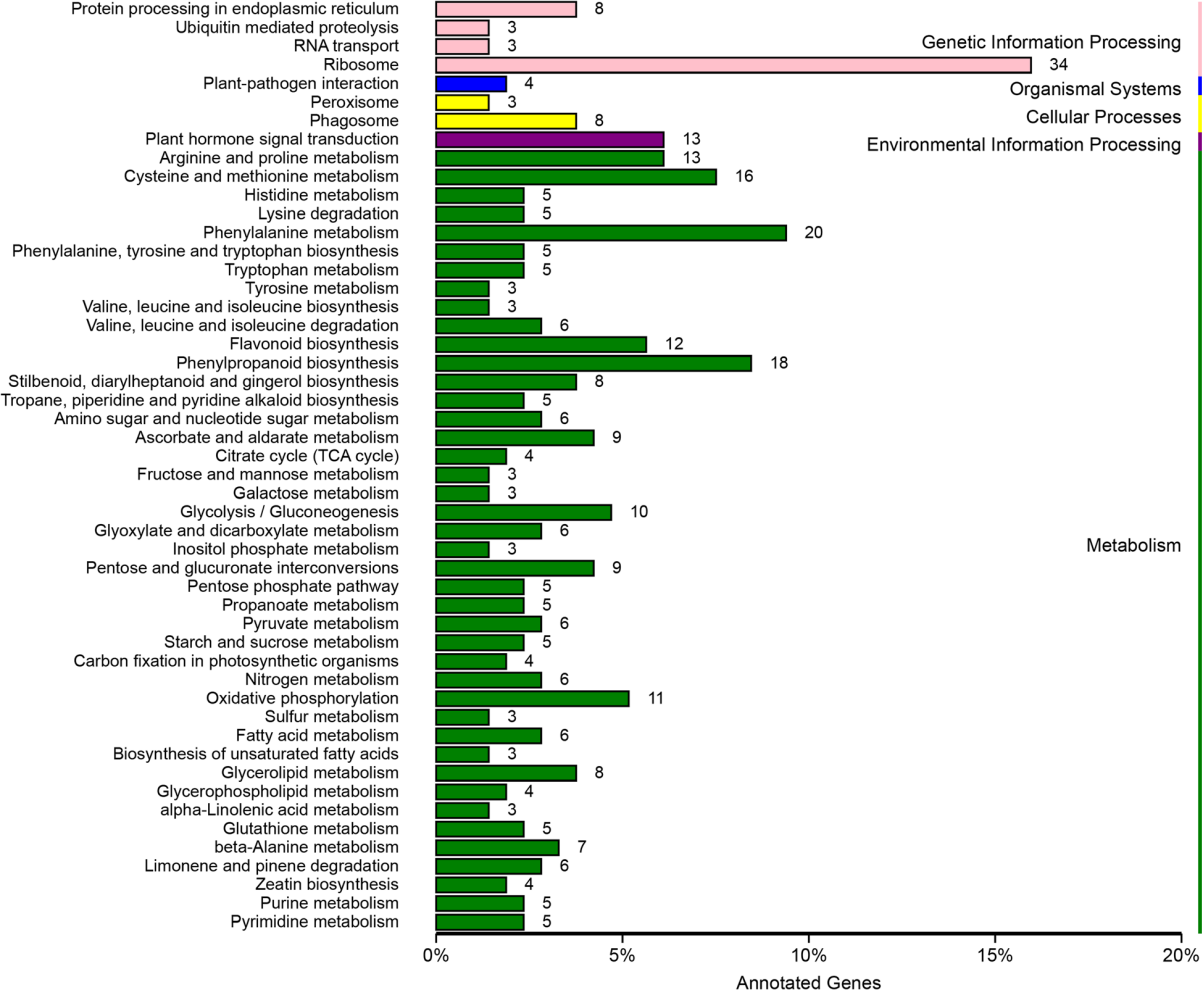
Gene ontology (GO) analysis of DEGs identified in TGY (Wei) *vs*. TGY (Wang)

In the TGY (Wei) *vs*. TGY (Wang) comparison, the DEGs could be divided into four major categories: metabolism, genetic information processing, environmental information processing, and organismal systems (Fig. 4). The metabolism category had the highest number of DEGs. Most of the enriched pathways were involved in metabolism and biosynthesis. The top 10 enriched pathways were stilbenoid, diarylheptanoid, and gingerol biosynthesis; limonene and pinene degradation; flavone and flavonol biosynthesis; flavonoid biosynthesis; phenylalanine metabolism; tropane, piperidine, and pyridine alkaloid biosynthesis; phenylpropanoid biosynthesis; zeatin biosynthesis; ascorbate and aldarate metabolism; and lysine degradation.

**Fig. 5:**
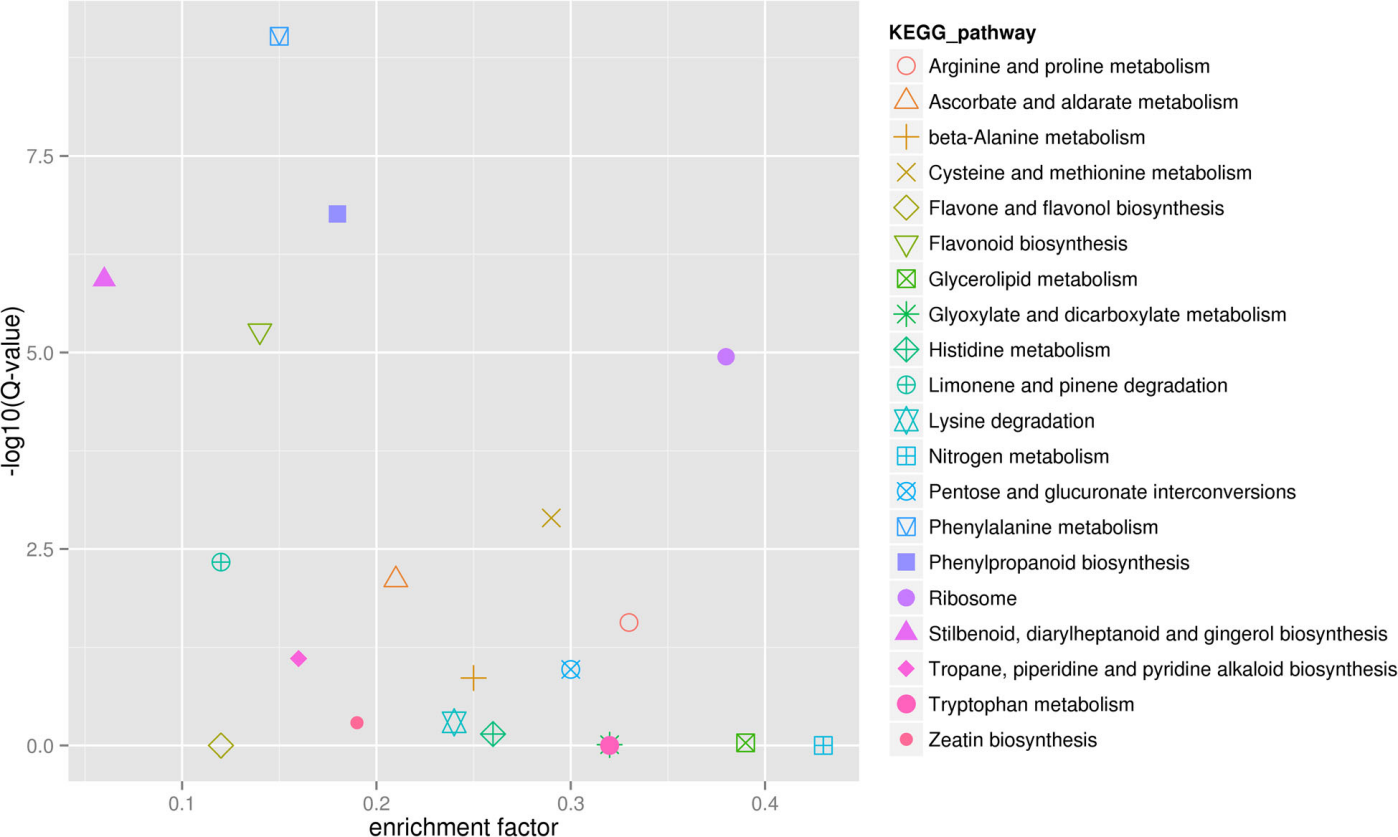
Kyoto Encyclopedia of Genes and Genomes (KEGG) enrichment analyses of differentially expressed genes (DEGs) identified in TGY (Wei) *vs*. TGY (Wang). Large enrichment factor represents high degree of enrichment. Lower q-value represents more significant enrichment of DEGs

The pathways with the higher enrichment rankings were the flavonoid biosynthesis pathway; the tropane, piperidine and pyridine alkaloid biosynthesis pathway, and the limonene and pinene degradation pathway (Fig. 5). Considering the effects of flavonoids and caffeine on tea taste and the effects of terpenoids on tea aroma, and the results of the KEGG analysis of DEGs in TGY (Wei) *vs*. TGY (Wang), we focused on the flavonoid, caffeine, and limonene metabolic pathways for further analyses.

**Fig. 6:**
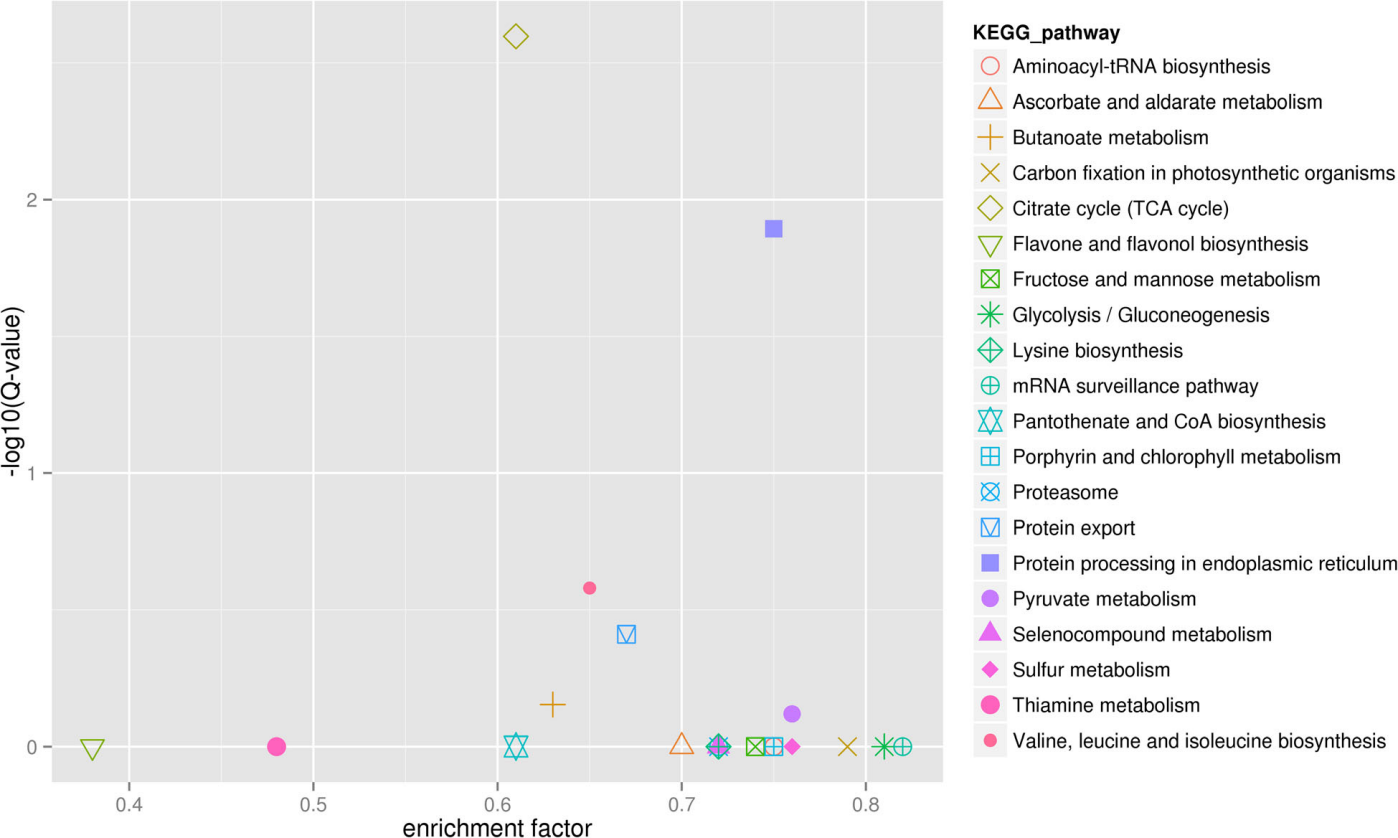
Kyoto Encyclopedia of Genes and Genomes (KEGG) enrichment analyses of differentially expressed genes (DEGs) identified in TGY (Wei) *vs*. BS. Large enrichment factor represents high degree of enrichment. Lower q-value represents more significant enrichment of DEGs

**Fig. 7:**
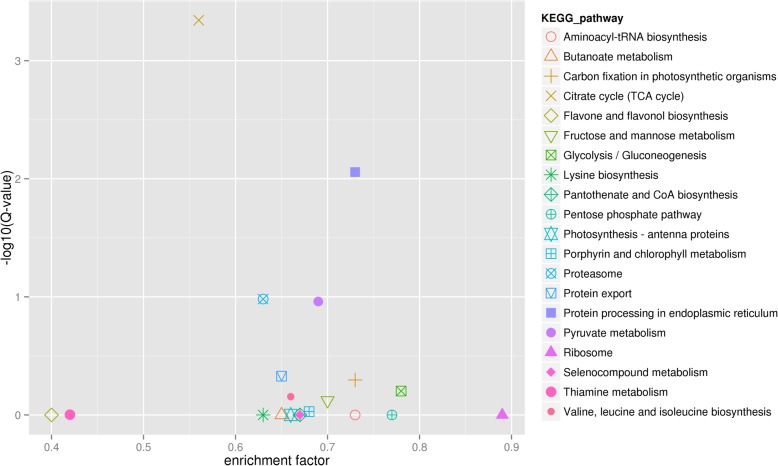
Kyoto Encyclopedia of Genes and Genomes (KEGG) enrichment analyses of differentially expressed genes (DEGs) identified in TGY (Wang) *vs*. BS. Large enrichment factor represents high degree of enrichment. Lower q-value represents more significant enrichment of DEGs

In the TGY (Wei) *vs*. BS and TGY (Wang) *vs*. BS comparisons, six out of the top 10 enriched pathways were consistent (Figs. 6 and 7). These six pathways were flavone and flavonol biosynthesis; thiamine metabolism; citrate cycle (TCA cycle); butanoate metabolism; valine, leucine, and isoleucine biosynthesis; and protein export. The flavone and flavonol biosynthesis pathway had the higher ranking in both comparisons. Based on these results, combined with the differences in leaf color and sensory quality between TGY and BS tea, we selected pigment-related and tea quality-related flavonoid metabolic pathways for further analyses.

### Analysis of DEGs involved in flavonoid metabolic pathways in ‘Tieguanyin’ (Wei), ‘Tieguanyin’ (Wang), and ‘Benshan’

**Fig. 8:**
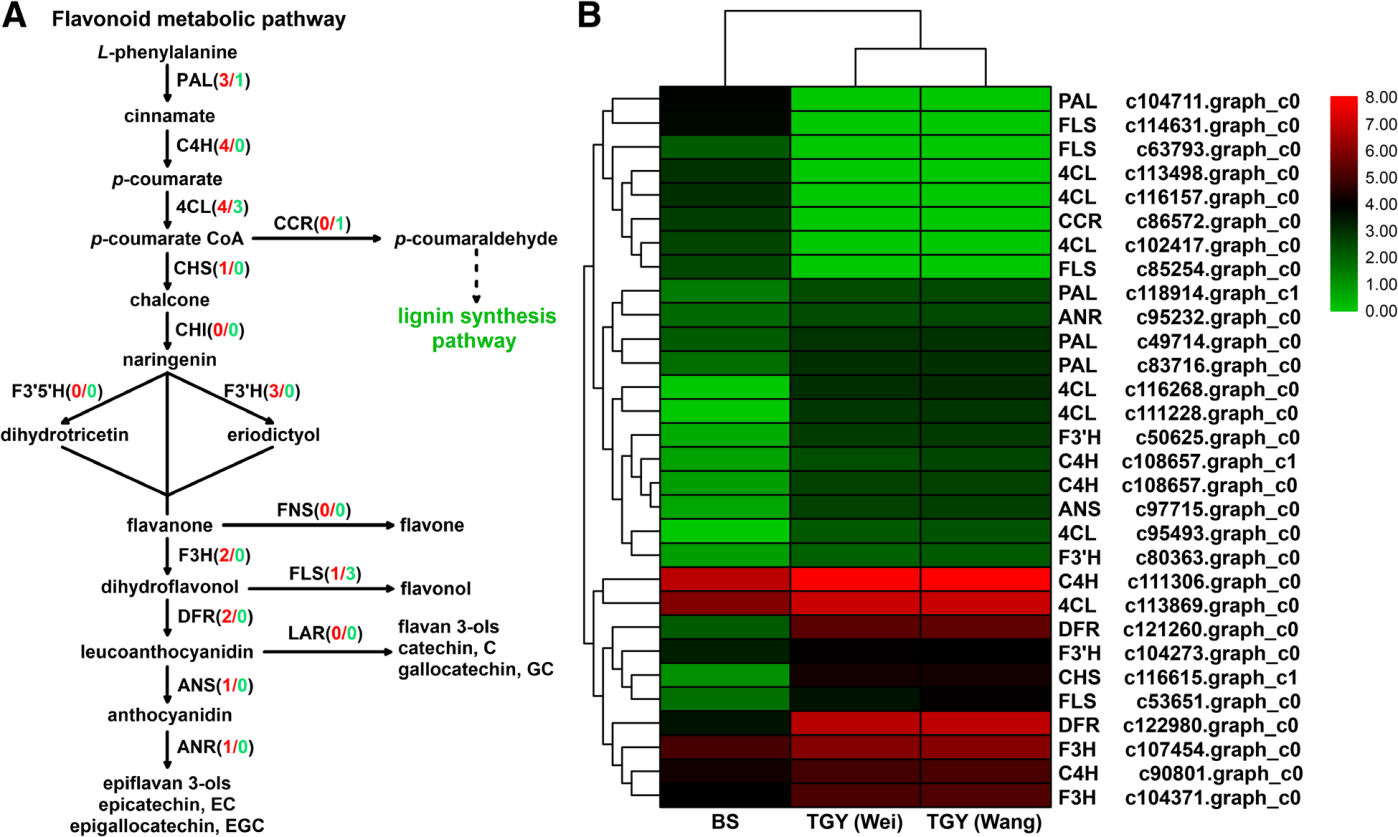
Differentially expressed genes (DEGs) involved in flavonoid metabolic pathway in TGY and BS. **a** Flavonoid metabolic pathway. Genes in red font were highly expressed in TGY; genes in green font were highly expressed in BS. Numbers in parentheses following each gene name indicate number of corresponding DEGs. **b** Hierarchical clustering analysis of relative expression levels of DEGs related to flavonoid metabolic pathway

To further understand the expression of DEGs involved in flavonoid metabolic pathways in TGY (Wei), TGY (Wang), and BS, we focused on the 30 differentially expressed unigenes annotated to this pathway. More than two unigenes corresponded to *PAL* (encoding phenylalanine ammonia-lyase), *C4H* (encoding 4-coumarate CoA ligase), *F3H* (encoding flavanone 3-hydroxylase), *FLS* (encoding flavonol synthase), *DFR* (encoding dihydroflavonol 4-reductase), and *F3’H* (encoding flavonoid 3′-hydroxylase) (Table 4). Only one unigene corresponded to *CHS* (encoding chalcone synthase), *ANS* (encoding anthocyanidin synthase), *ANR* (encoding anthocyanidin reductase), and *CCR* (encoding cinnamoyl-CoA reductase). On the basis of these identified DEGs, a proposed flavonoid metabolic pathway was constructed (Fig. 8a).

**Table 4:** Differentially expressed genes (DEGs) related to flavonoid metabolic pathway in TGY (Wei), TGY (Wang), and BS

Gene ID	KO ID	Annotation
c118914.graph_c1	K10775	phenylalanine ammonia-lyase (PAL)
c49714.graph_c0	K10775	phenylalanine ammonia-lyase (PAL)
c83716.graph_c0	K10775	phenylalanine ammonia-lyase (PAL)
c104711.graph_c0	K10775	phenylalanine ammonia-lyase (PAL)
c90801.graph_c0	K00487	cinnamate 4-hydroxylase (C4H)
c111306.graph_c0	K00487	cinnamate 4-hydroxylase (C4H)
c108657.graph_c0	K00487	cinnamate 4-hydroxylase (C4H)
c108657.graph_c1	K00487	cinnamate 4-hydroxylase (C4H)
c113869.graph_c0	K01904	4-coumarate CoA ligase (4CL)
c116268.graph_c0	K01904	4-coumarate CoA ligase (4CL)
c111228.graph_c0	K01904	4-coumarate CoA ligase (4CL)
c95493.graph_c0	K10526	4-coumarate CoA ligase (4CL)
c113498.graph_c0	K10526	4-coumarate CoA ligase (4CL)
c102417.graph_c0	K10526	4-coumarate CoA ligase (4CL)
c116157.graph_c0	K10526	4-coumarate CoA ligase (4CL)
c116615.graph_c1	K00660	chalcone synthase (CHS)
c107454.graph_c0	K09755	flavanone 3-hydroxylase (F3H)
c104371.graph_c0	K00475	flavanone 3-hydroxylase (F3H)
c53651.graph_c0	K05278	flavonol synthase (FLS)
c63793.graph_c0	K06892	flavonol synthase (FLS)
c114631.graph_c0	K06892	flavonol synthase (FLS)
c85254.graph_c0	K06892	flavonol synthase (FLS)
c121260.graph_c0	K13082	dihydroflavonol 4-reductase (DFR)
c122980.graph_c0	K13082	dihydroflavonol 4-reductase (DFR)
c50625.graph_c0	K00475	flavonoid 3′-hydroxylase (F3’H)
c104273.graph_c0	K05280	flavonoid 3′-hydroxylase (F3’H)
c80363.graph_c0	K05280	flavonoid 3′-hydroxylase (F3’H)
c97715.graph_c0	K05277	anthocyanidin synthase (ANS)
c95232.graph_c0	K08695	anthocyanidin reductase (ANR)
c86572.graph_c0	K09753	cinnamoyl-CoA reductase (CCR)

Comparisons of the transcript levels (FPKM values) of these DEGs revealed no significant differences in their transcript levels between TGY (Wei) and TGY (Wang) (Fig. 8b). However, in the TGY (Wei) *vs*. BS and TGY (Wang) *vs*. BS comparisons, more than half of these 30 unigenes showed higher transcript levels in TGY (Wei) and TGY (Wang) than in BS. The genes *PAL*, *C4H*, *CHS*, *F3’H*, *F3H*, *DFR*, *ANS*, and *ANR* had higher transcript levels in TGY (Wei) and TGY (Wang) than in BS. Seven of the unigenes corresponding to *4CL*, *FLS*, and *CCR* had lower transcript levels in TGY (Wei) and TGY (Wang) than in BS. These results implied that flavonoid metabolism had a stronger metabolic flux in TGY (Wei) and TGY (Wang) than in BS. The transcript level of *FLS* may be positively correlated with flavonol biosynthesis, but negatively correlated with anthocyanin and catechins accumulation. Hence, the high transcript level of *FLS* in BS may affect the accumulation of flavanols, anthocyanin, and catechins.

**Fig. 9:**
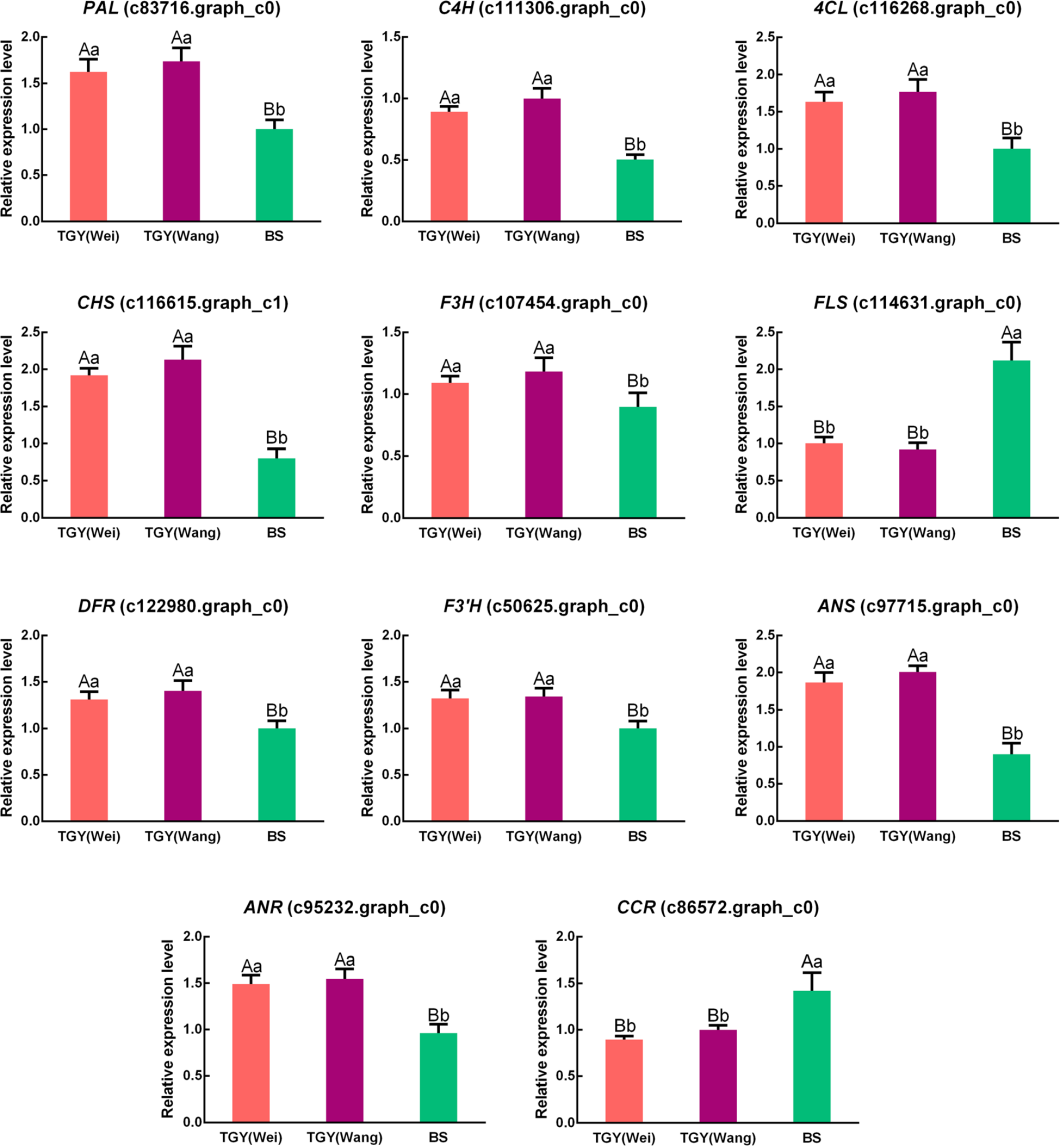
Expression patterns of differentially expressed genes (DEGs) in flavonoid metabolic pathway as determined by qRT-PCR. Data are means ± standard deviations (SD). Lowercase letter indicates significant difference (*P* < 0.05); uppercase letter indicates highly significant difference (*P* < 0.01)

To confirm the gene expression patterns detected in the transcriptome dataset, we conducted qRT-PCR analyses to determine the transcript levels of *PAL* (c83716.graph_c0), *C4H* (c111306.graph_c0), *4CL* (c116268.graph_c0), *CHS* (c116615.graph_c1), *F3H* (c107454.graph_c0), *FLS* (c114631.graph_c0), *DFR* (c122980.graph_c0), *F3’H* (c50625.graph_c0), *ANS* (c97715.graph_c0), *ANR* (c95232.graph_c0), and *CCR* (c86572.graph_c0) in TGY (Wei), TGY (Wang), and BS (Fig. 9). The expression trends of these unigenes detected in the qRT-PCR analyses were consistent with those detected in the RNA-seq dataset.

### Analysis of DEGs involved in caffeine metabolic pathway in ‘Tieguanyin’ (Wei), ‘Tieguanyin’ (Wang), and ‘Benshan’

**Fig. 10:**
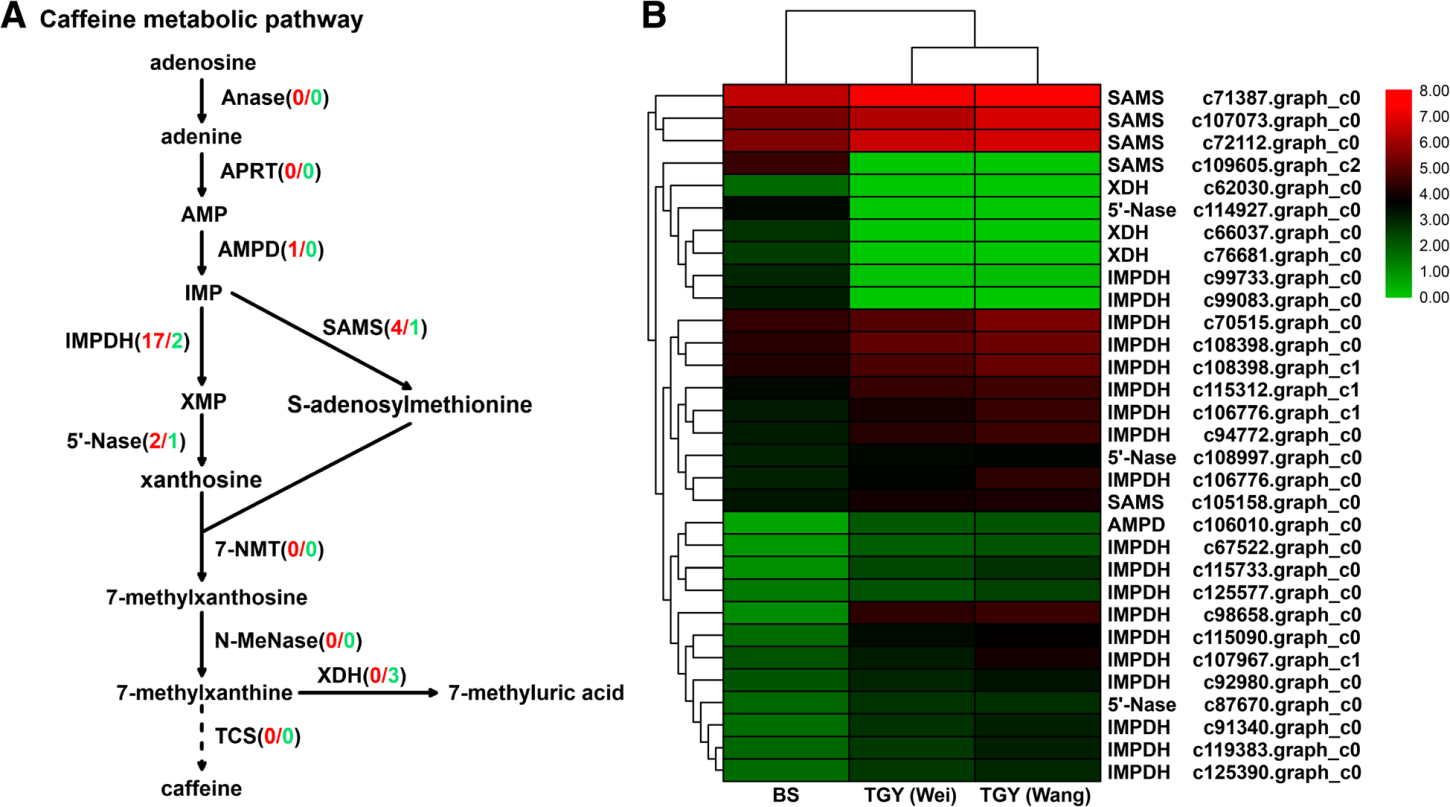
Differentially expressed genes (DEGs) involved in caffeine metabolic pathway in TGY and BS. **a** Caffeine metabolic pathway. Genes in red font were highly expressed in TGY; genes in green font were highly expressed in BS. Numbers in parentheses following each gene name indicate number of corresponding DEGs. **b** Hierarchical clustering analysis of relative expression levels of DEGs related to caffeine metabolic pathway

The KEGG analysis indicated that the caffeine metabolic pathway was one of the representative pathways in these tea plants. A total of 31 differentially expressed unigenes were assigned to genes in the caffeine metabolic pathway. Among them, only one unigene corresponded to *AMPD* (encoding AMP deaminase), while more than one unigene corresponded to *IMPDH* (encoding IMP dehydrogenase), *SAMS* (encoding S-adenosylmethionine synthase), *5*′*-Nase* (encoding 5′-nucleotidase), and *XDH* (encoding xanthine dehydrogenase). The *IMPDH* gene had the largest number of corresponding unigenes (Table 5). On the basis of these identified DEGs, a proposed caffeine metabolic pathway was constructed (Fig. 10a).

**Table 5:** Differentially expressed genes (DEGs) related to caffeine metabolic pathway in TGY (Wei), TGY (Wang), and BS

Gene ID	KO ID	Annotation
c106010.graph_c0	K01490	AMP deaminase (AMPD)
c108398.graph_c0	K10999	IMP dehydrogenase (IMPDH)
c108398.graph_c1	K10999	IMP dehydrogenase (IMPDH)
c106776.graph_c0	K10999	IMP dehydrogenase (IMPDH)
c115312.graph_c1	K10999	IMP dehydrogenase (IMPDH)
c70515.graph_c0	K10999	IMP dehydrogenase (IMPDH)
c106776.graph_c1	K10999	IMP dehydrogenase (IMPDH)
c94772.graph_c0	K10999	IMP dehydrogenase (IMPDH)
c115090.graph_c0	K00088	IMP dehydrogenase (IMPDH)
c115733.graph_c0	K14493	IMP dehydrogenase (IMPDH)
c98658.graph_c0	K14493	IMP dehydrogenase (IMPDH)
c125577.graph_c0	K10999	IMP dehydrogenase (IMPDH)
c119383.graph_c0	K10999	IMP dehydrogenase (IMPDH)
c91340.graph_c0	K10999	IMP dehydrogenase (IMPDH)
c67522.graph_c0	K10999	IMP dehydrogenase (IMPDH)
c107967.graph_c1	K10999	IMP dehydrogenase (IMPDH)
c92980.graph_c0	K10999	IMP dehydrogenase (IMPDH)
c125390.graph_c0	K10999	IMP dehydrogenase (IMPDH)
c99733.graph_c0	K10999	IMP dehydrogenase (IMPDH)
c99083.graph_c0	K10999	IMP dehydrogenase (IMPDH)
c107073.graph_c0	K00789	S-adenosylmethionine synthase (SAMS)
c71387.graph_c0	K00789	S-adenosylmethionine synthase (SAMS)
c72112.graph_c0	K00789	S-adenosylmethionine synthase (SAMS)
c105158.graph_c0	K00789	S-adenosylmethionine synthase (SAMS)
c109605.graph_c2	K00789	S-adenosylmethionine synthase (SAMS)
c87670.graph_c0	K03787	5′-nucleotidase (5′-Nase)
c108997.graph_c0	K00326	5′-nucleotidase (5′-Nase)
c114927.graph_c0	K03787	5′-nucleotidase (5′-Nase)
c62030.graph_c0	K00106	Xanthine dehydrogenase (XDH)
c66037.graph_c0	K00106	Xanthine dehydrogenase (XDH)
c76681.graph_c0	K00106	Xanthine dehydrogenase (XDH)

The expression trends of the 31 differentially expressed unigenes involved in caffeine metabolism were compared based on transcriptome FPKM values (Fig. 10b). In the TGY (Wei) *vs*. TGY (Wang) comparison, except for two unigenes corresponding to *IMPDH* and *SAMS*, the key caffeine metabolic genes showed no significant difference in their expression levels between the two TGY samples.

In the TGY (Wei) *vs*. BS and TGY (Wang) *vs*. BS comparisons, most genes involved in the caffeine metabolic pathway had higher transcript levels in TGY than in BS. Four genes, *AMPD* (1 unigene), *IMPDH* (17 unigenes), *SAMS* (4 unigenes), *5′-Nase* (2 unigenes) had higher transcript levels in TGY (Wei) and TGY (Wang) than in BS. Increased transcript levels of *AMPD*, *IMPDH*, *SAMS* and *5′*-*Nase* genes may be positively correlated with the amount of precursors for caffeine metabolism. However, the up-regulation of *XDH* in BS could result in competition for precursors for caffeine biosynthesis and could also affect caffeine accumulation.

**Fig. 11:**
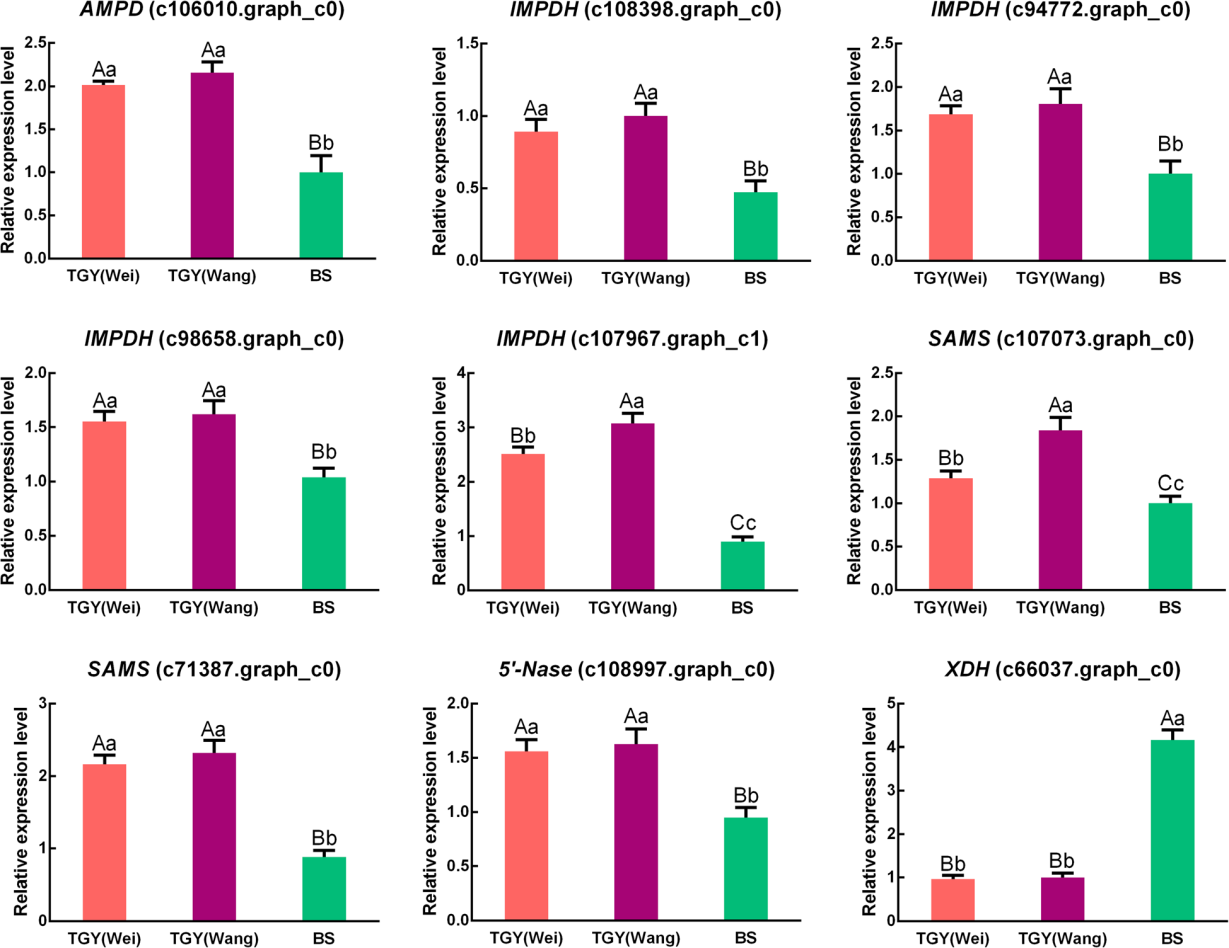
Expression patterns of differentially expressed genes (DEGs) in caffeine metabolic pathway as determined by qRT-PCR. Data are means ± standard deviations (SD). Lowercase letter indicates significant difference (*P* < 0.05); uppercase letter indicates highly significant difference (*P* < 0.01)

To confirm the gene expression trends detected in the transcriptome dataset, we conducted qRT-PCR analyses to detect the transcript levels of *AMPD* (c106010.graph_c0), *IMPDH* (c108398.graph_c0, c94772.graph_c0, c98658.graph_c0 and c107967.graph_c1), *SAMS* (c107073.graph_c0 and c71387.graph_c0), *5′-Nase* (c108997.graph_c0), and *XDH* (c66037.graph_c0) in TGY (Wei), TGY (Wang), and BS (Fig. 11). The trends in gene expression were similar between the qRT-PCR and RNA-seq analyses. Together, these results confirmed that there were transcriptional-level differences in the caffeine metabolic pathway and caffeine biosynthesis between TGY and BS.

### Analyses of DEGs involved in limonene degradation pathway in ‘Tieguanyin’ (Wei), ‘Tieguanyin’ (Wang), and ‘Benshan’

**Fig. 12:**
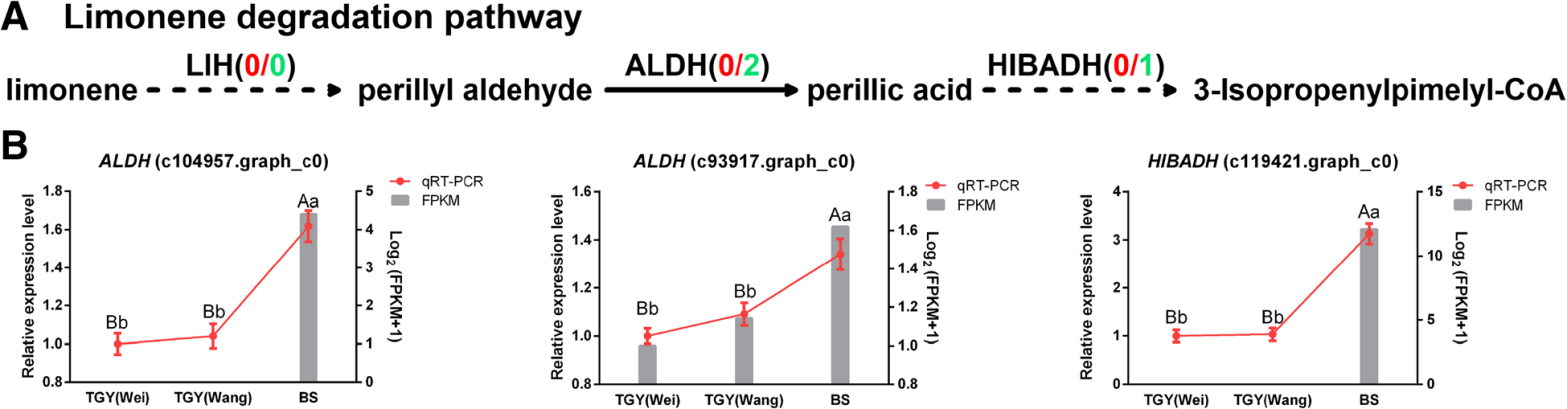
Differentially expressed genes (DEGs) involved in limonene degradation pathway in TGY and BS. **a** Limonene degradation pathway. Genes in red font were highly expressed in TGY; genes in green font were highly expressed in BS. **b** Expression patterns of DEGs in caffeine metabolic pathway as determined by qRT-PCR. Data are means ± standard deviations (SD). Lowercase letter indicates significant difference (*P* < 0.05); uppercase letter indicates highly significant difference (*P* < 0.01)

Next, we identified unigenes corresponding to genes in the limonene degradation pathway in the transcriptome data. Three unigenes encoding two enzymes were identified by mapping to the KEGG pathway. One unigene corresponded to *HIBADH*, encoding hydroxyisobutyrate dehydrogenase, and more than one unigene corresponded to *ALDH*, encoding aldehyde dehydrogenase (Additional file 9: Table S4). A proposed limonene degradation pathway was constructed on the basis of these identified DEGs (Fig. 12a).

The expression trends of these three unigenes were compared among TGY (Wei), TGY (Wang), and BS based on FPKM values (Fig. 12b). There was no significant difference in the expression levels of *ALDH* and *HIBADH* between TGY (Wei) and TGY (Wang), but both of these genes were significantly up-regulated in BS *vs*. TGY (Wei) and BS *vs*. TGY (Wang). The expression levels of *ALDH* and *HIBADH* are known to be negatively correlated with limonene content. Thus, high transcript levels of these two genes may lead to greater decomposition of limonene in BS than in TGY (Wei) and TGY (Wang). In other words, more limonene may be retained in TGY. A previous report indicated that limonene plays a significant role in tea flavor [36], so it may be a key factor in the “Yin Rhyme” flavor of TGY.

To confirm the gene expression trends detected in the transcriptome dataset, we conducted qRT-PCR analyses to determine the transcript levels of *ALDH* (c104957.graph_c0), *ALDH* (c93917.graph_c0), and *HIBADH* (c119421.graph_c0) in TGY (Wei), TGY (Wang), and BS (Fig. 12b). The expression patterns of the detected unigenes were consistent with the RNA-seq results. The gene expression patterns were also consistent with the differences in limonene contents among the tea plants. Thus, limonene accumulation may be a factor contributing to the unique “Yin Rhyme” flavor of TGY.

### Analyses of CsPAP1 TF involved in flavonoid and anthocyanin biosynthesis in ‘Tieguanyin’ (Wei), ‘Tieguanyin’ (Wang), and ‘Benshan’

The PAP1 TF has been shown to play roles in controlling flavonoid and anthocyanin biosynthesis in *Arabidopsis thaliana* [37] and rose [38]. However, the relationship between *CsPAP1* expression and anthocyanin accumulation in tea is unclear. In our transcriptome data, among all the TF genes, *PAP1* had the largest difference in FPKM values in the TGY (Wei) *vs*. BS and TGY (Wang) *vs*. BS comparisons. Therefore, we analyzed CsPAP1 in more detail. Using the HMM file of the PAP1 domain, we searched the tea transcriptome data using HMMER 3.0 software, and obtained candidate CsPAP1 TFs. The protein sequences of the candidate CsPAP1 TFs were further confirmed using tools at the InterPro database. We finally identified five unigenes corresponding to *CsPAP1* (c65777.graph_c0, c112219.graph_c0, c53572.graph_c0, c121522.graph_c0, and c124394.graph_c0) in TGY (Wei), TGY (Wang), and BS.

**Fig. 13:**
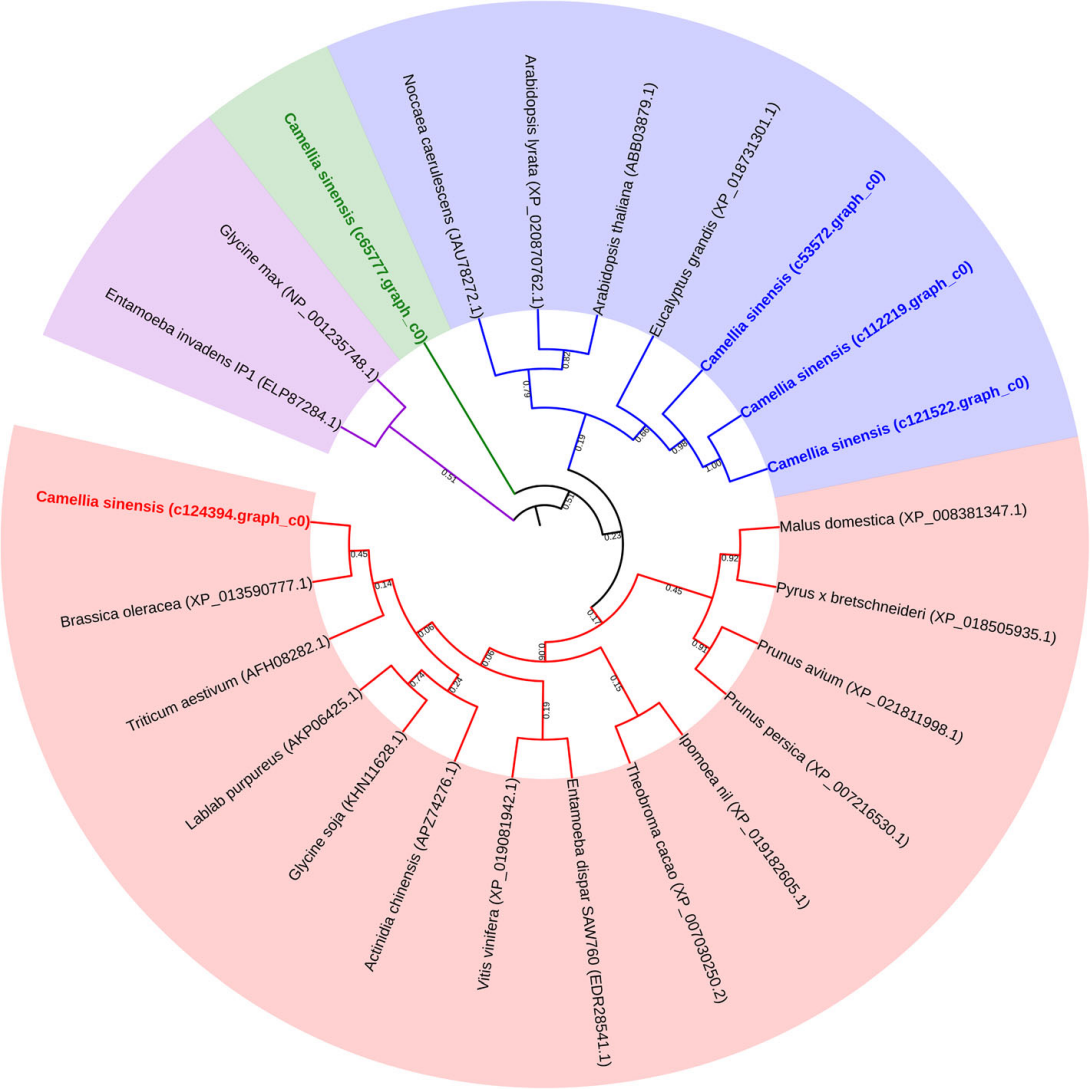
Evolutionary relationships of *PAP1* genes in different plants

In the phylogenetic tree (Fig. 13), the five CsPAP1s were distributed in three clusters: one cluster contained three CsPAP1 sequences (c53572.graph_c0, c112219.graph_c0, and c121522.graph_c0), while the two other clusters contained c65777.graph_c0 and c124394.graph_c0. The c65777.graph_c0 was in a cluster genetically distant from the other four CsPAP1. The c124394.graph_c0 clustered with PAP1 from *Brassica oleracea*, but was separate from three tea CsPAP1s (c53572.graph_c0, c112219.graph_c0, and c121522.graph_c0). Among those three tea CsPAP1s, c112219.graph_c0 and c121522.graph_c0 had closer genetic distances with each other than with c53572.graph_c0.

**Fig. 14:**
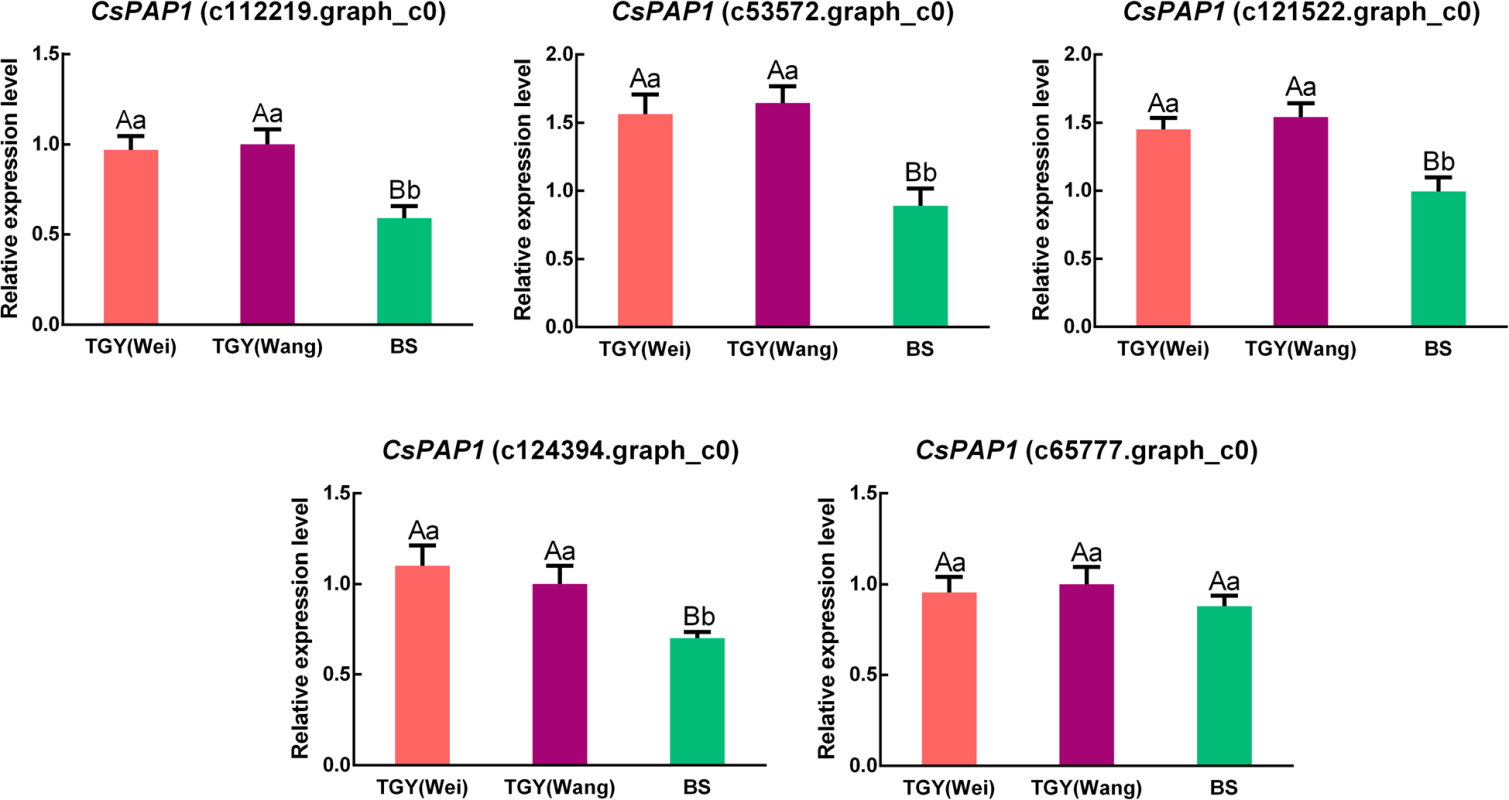
Expression patterns of *CsPAP1* as determined by qRT-PCR. Data are means ± standard deviations (SD). Lowercase letter indicates significant difference (*P* < 0.05); uppercase letter indicates highly significant difference (*P* < 0.01)

The expression patterns of the five *CsPAP1* genes were analyzed by qRT-PCR in TGY (Wei), TGY (Wang), and BS (Fig. 14). Four of the *CsPAP1* genes (c112219.graph_c0, c53572.graph_c0, c121522.graph_c0, and c124394.graph_c0) had higher transcript levels in TGY (Wei) and TGY (Wang) than in BS. The other one, c65777.graph_c0, had slightly higher transcript levels in TGY (Wei) and TGY (Wang) than in BS.

A functional prediction analysis of the CsPAP1s showed that c112219.graph_c0, c53572.graph_c0, and c121522.graph_c0 may promote the synthesis of phenylpropanoid-derived compounds such as anthocyanin and catechins, probably together with ENHANCER OF GLABRA3 (EGL3) and bHLH. There may be a positive correlation between the expression of PAP1 and the accumulation of anthocyanin and catechins. Therefore, differences in the transcript levels of *CsPAP1* genes may explain differences in catechins and anthocyanin contents between TGY and BS.

## Discussion

### Differences in anthocyanin and carotenoids contents may be responsible for deeper purple leaf color of ‘Tieguanyin’ than ‘Benshan’

Chlorophylls, anthocyanin, and carotenoids are the main plant pigments that affect leaf color formation in higher plants. Differences in leaf color among different cultivars are due to the relative contents of three main pigment groups (chlorophylls, carotenoids, and anthocyanin) [39–42]. Previous analyses of biological characteristics have confirmed that the leaf color of BS is green, while those of the two TGY plants are purple. However, the main pigment components and their contents in these tea plants had not been determined. Therefore, we determined the chlorophylls, carotenoids, and anthocyanin contents in TGY (Wang), TGY (Wei), and BS. The anthocyanin contents were higher in TGY (Wei) and TGY (Wang) than in BS. Similarly, the chlorophylls and carotenoids contents were higher in TGY than in BS. High concentrations of anthocyanin are responsible for the red, blue, and purple colors of plant leaves [43]. Carotenoids, which are synthesized by a variety of enzymes in plastids, are responsible for yellow and red colors in leaves [44]. The original TGY tea plants had high chlorophylls contents but still had a purple leaf color. Therefore, we inferred that anthocyanin and carotenoids at sufficiently high concentrations can mask the green color of chlorophylls. The results indicated that the leaf color of TGY mainly depends on high levels of anthocyanin and carotenoids. These observations were consistent with those of a previous study on *Quercus coccifera* [45]. Together, these results indicated that high contents of anthocyanin and carotenoids, but not chlorophylls, play critical roles in the purple leaf color of TGY (Wei) and TGY (Wang).

Previous studies found that the leaves of the tea cultivar ‘Ziyan’ [46] and eucalyptus [47] with high concentrations of anthocyanin also contained high levels of phenolic compounds. The results of this study suggested that TGY (Wei) and TGY (Wang), which had high anthocyanin contents, may also be rich in other flavonoid substances, especially catechins. This may contribute to the high quality of TGY tea.

### Combined with the expression levels of genes related to secondary metabolism, high contents of catechins, anthocyanin, caffeine, and limonene contribute to unique “Yin Rhyme” flavor in ‘Tieguanyin’

In this study, compared with leaves of BS, the leaves of TGY (Wei) and TGY (Wang) had higher levels of catechins, caffeine, and limonene. However, there were no significant differences in the levels of these three metabolites between TGY (Wei) and TGY (Wang). To explore the mechanism underlying the high contents of these metabolites in TGY, we conducted pathway enrichment analyses. The results of KEGG analyses indicated that the flavonoid, caffeine, and limonene metabolic pathways were the representative pathways in these tea cultivars. In total, 64 differentially expressed unigenes were assigned to these three metabolic pathways. The expression patterns of these unigenes were analyzed by qRT-PCR in TGY (Wei), TGY (Wang), and BS. The transcript levels of *PAL*, *C4H*, *CHS*, *F3’H*, *F3H*, *DFR*, *ANS*, and *ANR,* all of which are involved in the flavonoid metabolic pathway, were up-regulated in TGY (Wei) and TGY (Wang) compared with BS. Two genes (*FLS* and *CCR*) showed higher transcript levels in BS than in TGY (Wei) and TGY (Wang). In the caffeine metabolism pathway, *AMPD*, *IMPDH*, *SAMS,* and *5′-Nase* had higher transcript levels in TGY (Wei) and TGY (Wang) than in BS, but *XDH* had lower transcript levels in TGY (Wei) and TGY (Wang) than in BS. Two genes in the limonene degradation pathway, *ALDH* and *HIBADH*, were down-regulated in TGY (Wei) *vs*. BS and TGY (Wang) *vs*. BS.

Eight genes in the flavonoid and anthocyanin biosynthesis pathways (*PAL*, *C4H*, *CHS*, *F3’H*, *F3H*, *DFR*, *ANS*, and *ANR*) had higher transcript levels in TGY than in BS. PAL is the first limiting enzyme in the flavonoid metabolic pathway and its product is a direct or indirect precursor of many secondary metabolites [46, 47]. Many studies have shown that *PAL* expression is significantly positively correlated with flavonoid and anthocyanin accumulation, indicating that it plays a critical role in regulating the production of secondary metabolites [48–50]. Its high expression directs metabolic flux into flavonoid biosynthesis to define the size of the anthocyanin and catechins pool [51]. There was a positive relationship between *PAL* expression levels and anthocyanin and catechins contents in the two TGY tea plants, and these compounds were present at significantly higher concentrations in TGY than in BS. The second step of the flavonoid metabolic pathway is catalyzed by C4H. In *Arabidopsis*, *AtC4H* and *AtPAL1* were found to have similar expression patterns, both in terms of tissue-specific expression and expression under stress treatments. The expression patterns of these two genes were shown to be positively correlated [52]. The promoter of *AtC4H* contains the same three *cis*-acting elements, like that of *AtPAL* [53]. Liu et al. reported that the flavonoid and catechins contents in tea leaves were closely related to *C4H* expression [54]. Consistent with this, we observed that the transcript levels of *C4H* and catechins contents were significantly higher in TGY (Wei) and TGY (Wang) than in BS.

In the flavonoid synthesis pathway, F3’H catalyzes the conversion of naringenin and dihydrokaempferol into eriodictyol and dihydroquercetin, which are important intermediates for the biosynthesis of anthocyanin and catechins [55, 56]. A previous study revealed a strong positive correlation between the transcript levels of *F3’H* and catechins contents in tea plants [56]. Another key enzyme in the flavonoid metabolic pathway is F3H. High transcript levels of *F3H* were found to promote the accumulation of anthocyanin and flavonoids [57–59]. This enzyme co-ordinates with CHS and CHI [60], while DFR, ANS, and ANR play important roles in the biosynthesis of anthocyanin and catechins [61–63]. Vinay et al. found that the overexpression of *CsDFR* and *CsANR* in tobacco led to improved flavonoid contents, especially total catechins, EC, and EGC. Other studies have reported that *DFR* and *ANS* are key genes for increasing the catechins and anthocyanin contents in *C. sinensis* [64, 65]. We found that high transcript levels of *DFR*, *ANS*, and *ANR* were positively correlated with anthocyanin and catechins contents in TGY (Wei) and TGY (Wang). Together, our results showed that up-regulation of these eight genes in the flavonoid metabolic pathway was positively correlated with the accumulation of catechins and anthocyanin. The high transcript levels of these genes may be the key factors in the formation of the “Yin Rhyme” flavor of TGY.

A key enzyme in lignin synthesis is CCR, and its abundance directly affects the metabolism of lignin and many phenolic substances in plants [66]. The lignin biosynthesis pathway is located downstream of, and is a branching point in, the phenylpropanoid metabolic pathway. It has parallel status with other branching pathways of phenylpropanoid metabolism such as the flavonoid metabolic pathway. A previous study showed that a high expression level of *CCR* is positively correlated with lignin production, while down-regulation of *CCR* may lead to reduced lignin content [67]. Therefore, the higher transcript level of *CCR* in BS than in TGY may shift the metabolic flux to lignin synthesis, away from flavonoid synthesis. This would reduce the amount of precursors for catechins synthesis in BS, which may explain the significantly lower catechins content in BS than in TGY.

Downstream of flavonoid synthesis, FLS is a branching enzyme whose activity is related to the synthesis of flavonols and flavan-3-ols [68–71]. A recent report on the draft genome of *C. sinensis* var. *sinensis* noted that high expression levels of *FLS* were positively correlated with the accumulation of monomeric galloylated catechins [2]. In this study, upregulation of *FLS* was negatively correlated with anthocyanin, epicatechin, and epigallocatechin contents; thus, FLS limited the size of these metabolite pools. These results indicated that high transcript levels of *PAL*, *C4H*, *CHS*, *F3’H*, *F3H*, *DFR*, *ANS*, and *ANR* and low transcript levels of *CCR* and *FLS* in TGY (Wei) and TGY (Wang) play critical roles in flavonoid metabolism. Metabolite analyses revealed that the contents of catechins (EC, EGC, ECG, GCG, and EGCG), total catechins, and anthocyanin were significantly higher in TGY (Wei) and TGY (Wang) than in BS. The high contents of catechins and anthocyanin may be correlated with the expression levels of genes related to flavonoid metabolism. This relationship may be an important factor in the formation of the unique “Yin Rhyme” flavor in ‘Tieguanyin’.

Caffeine is an indole alkaloid present in tea and coffee. It is one of the three main components that affect the sensory quality of tea [72, 73]. In this study, the contents of caffeine were significantly higher in TGY than in BS. This result may be related to the expression of DEGs involved in the caffeine metabolic pathway.

Compared with BS, TGY showed up-regulated expression of most of the DEGs in the caffeine metabolic pathway. This result indicated that there was stronger metabolic flux into the caffeine metabolic pathway in TGY than in BS. In total, 23 unigenes in the tea transcriptome corresponded to *AMPD*, *IMPDH*, *SAMS*, and *5′-Nase*, which encode four vital enzymes in the caffeine metabolic pathway. These four genes had higher transcript levels in TGY (Wei) and TGY (Wang) than in BS. In tea plants, AMP deaminase is a key enzyme involved in caffeine biosynthesis, and it mainly catalyzes the deamination of adenosine to produce hypoxanthine nucleotides. Addition of an AMP deaminase inhibitor to tea flower buds was shown to reduce *AMPD* expression and caffeine content [74], indicating that the expression level of *AMPD* is positively correlated caffeine content. Another crucial enzyme is SAMS, which produces xanthine nucleosides and functions as the only methyl donor for methylation in caffeine metabolism. The reaction converting inosine monophosphate (IMP) into xanthosine monophosphate (XMP) is catalyzed by IMPDH, which promotes the synthesis of another caffeine precursor [75, 76]. Previous studies have shown that *SAMS* expression levels regulate the rate of SAM synthesis to provide sufficient precursors for the downstream synthesis of caffeine [76, 77]. Caffeine accumulation has also been found to be affected by *5*′*-Nase* expression levels [78]. Therefore, the up-regulated expression of these four genes may be positively correlated with caffeine content, which would explain the greater accumulation of caffeine in TGY (Wei) and TGY (Wang) than in BS.

The reason for the low caffeine content in BS may be the low transcript levels of *AMPD*, *IMPDH*, *SAMS*, *5′-Nase*, consistent with the results of previous studies [79, 80]. In addition, *XDH*, which encodes an intermediate enzyme in caffeine metabolism that catalyzes the formation of urate from xanthine and hypoxanthine [81, 82], was expressed at higher levels in BS than in TGY. The high expression of *XDH* may have steered the metabolic flow to 7-methyluric acid synthesis. We found that the transcript level of *XDH* was negatively correlated with the accumulation of precursors for caffeine synthesis. Previous studies on *Arabidopsis* revealed that caffeine accumulated to high concentrations after inhibition of *XDH* expression [83, 84], suggesting that caffeine content is negatively correlated with *XDH* expression. Based on these results, the high caffeine content in TGY may have been related to low *XDH* expression. Two previous studies on the tea genome [2, 22] suggested that some genes in the caffeine metabolic pathway have undergone expansion events, and that high expression of caffeine metabolism-related genes is positively correlated with caffeine accumulation. Those studies also suggested that high expression levels of flavonoid and caffeine metabolism-related genes, but not theanine metabolism-related genes, are the crucial factors for improving tea taste and aroma quality. Those results are consistent with our findings that the expression levels of four caffeine metabolism-related genes (*AMPD*, *IMPDH*, *SAMS*, and *5′-Nase* genes) were expressed at higher levels in TGY (Wei) and TGY (Wang) than in BS, but their expression levels did not differ significantly between TGY (Wei) and TGY (Wang). Combined with the caffeine content determination results, these results suggested that the high caffeine content in two original TGY tea plants may be one reason for unique “Yin Rhyme” flavor of TGY (Wei) and TGY (Wang).

Monoterpenoids are a class of terpenoids that are components of volatile aroma [35, 85]. Limonene is a monocyclic terpenoid that is a precursor for many other cyclic terpenoids; thus, it significantly affects terpenoid synthesis [86, 87]. Limonene is also an important component of tea aroma, and is mainly responsible for the floral fragrance of tea [35, 36].

We analyzed volatiles in TGY and BS, and found that except for limonene, the nine other main volatile compounds did not differ significantly in concentration between TGY and BS. To explore the differences in limonene between TGY and BS, we further analyzed the transcript level of genes related to this compound in the transcriptome data and by qRT-PCR. We found that *ALDH* and *HIBADH* were significantly up-regulated in BS *vs*. TGY (Wei) and BS *vs*. TGY (Wang). These genes encode enzymes that catalyze the decomposition of limonene into perillic acid and isopropenylpimelyl-CoA [88–90]. In BS, there were high transcript levels of these genes and a low limonene content. Lower expression levels of these genes in TGY would mean that less limonene would be degraded, which explains the significantly higher limonene content in TGY than in BS. Xie et al. found that the expression level of *ALDH* was inversely proportional to limonene content [91]. These results indicated that a low limonene content in BS may lead to a lack of sufficient precursor substances for the synthesis of cyclic terpenoids.

**Fig. 15:**
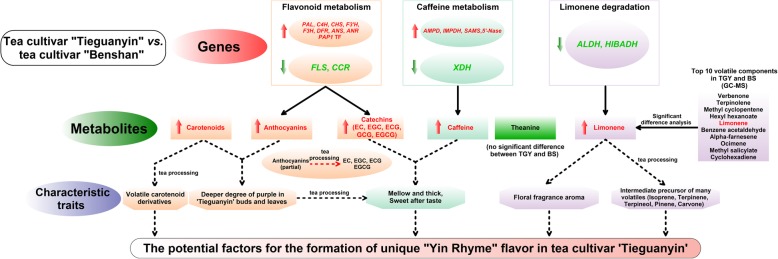
Schematic diagram of key genes and metabolites of flavonoid, caffeine, and limonene metabolism in tea cultivar ‘Tieguanyin’. Red font indicates gene with higher expression level or metabolite with higher content in TGY (Wei) and TGY (Wang) than in BS; green font indicates gene with lower expression level or metabolite with lower content in TGY (Wei) and TGY (Wang) than in BS. Solid line represents direct action; dashed line represents potential action

In the present study, compared with BS, TGY had eight up-regulated genes (*PAL*, *C4H*, *CHS*, *F3’H*, *F3H*, *DFR*, *ANS*, and *ANR*) and two down-regulated genes (*FLS* and *CCR*) in the flavonoid metabolic pathway; four up-regulated genes (*AMPD*, *IMPDH*, *SAMS*, and *5′*-*Nase*) and one down-regulated *XDH* gene in the caffeine metabolic pathway; and two down-regulated genes (*ALDH* and *HIBADH*) in the limonene degradation pathway. The expression patterns of these genes were related to the greater formation of catechins, anthocyanin, caffeine, and limonene in TGY (Wei) and TGY (Wang) than in BS. Combined with the expression levels of genes related to secondary metabolism, high contents of catechins, anthocyanin, caffeine, and limonene may be correlated with the formation of the “Yin Rhyme” flavor in ‘Tieguanyin’ (Fig. 15).

Apart from two unigenes corresponding to *IMPDH* (c107967.graph_c1) and *SAMS* (c107073.graph_c0), which are involved in caffeine metabolism, the expression levels of all other genes were not significantly different between TGY (Wei) and TGY (Wang). In addition, the contents of related metabolites, including flavonoids, anthocyanin, catechins, caffeine, and limonene, were not significantly different between the two TGY samples. Therefore, we inferred that the other unigenes corresponding to *IMPDH* and *SAMS* that were expressed at similar levels in TGY (Wei) and TGY (Wang) compensated for the differences in the expression of c107967.graph_c1 and c107073.graph_c0 between TGY (Wei) and TGY (Wang). On the basis of their similar secondary metabolite contents and expression levels of secondary metabolism-related genes, we speculated that these two original TGY plants may be sister strains of ‘Tieguanyin’, with similar tea aroma quality and the “Yin Rhyme” flavor.

### Up-regulated *CsPAP1* in ‘Tieguanyin’ compared with ‘Benshan’ promotes flavonoid and anthocyanin accumulation

A previous study has shown that the transcriptional regulation of structural genes is a major mechanism by which flavonoid and anthocyanin biosynthesis is regulated in plants [92]. In many plants, regulation occurs through a set of TFs including MYB, bHLH, and WD-repeat proteins. Members of the MYB family are often the major determinants of variation in anthocyanin pigmentation [93]. For example, *MYB114* has been proven to coordinately regulate anthocyanin biosynthesis via its interaction of *bHLH3* and *ERF3* in pear [94], and the R2R3-MYB TF anthocyanin 1 (*CsAN1*) regulates anthocyanin accumulation in purple-leaf tea [62]. PAP1 is a MYB TF that is involved in regulating flavonoid and anthocyanin biosynthesis [37]. Transgenic rose expressing *PAP1* not only showed a more intense purple color, but also had increased concentrations of terpenoid scent compounds in its flowers [38].

Interestingly, the TGY transcriptome dataset included unigenes corresponding to *PAP1* (GenBank accession: AF325123) with E-value < 1.0E^− 6^. The phylogenetic tree showed that CsPAP1 (c124394.graph_c0) clustered with PAP1 in *Brassica oleracea*, but deviated from the other three CsPAP1s (c112219.graph_c0, c53572.graph_c0, and c121522.graph_c0). Among these three CsPAP1s, c112219.graph_c0 and c121522.graph_c0 had a closer genetic distance with each other than with c53572.graph_c0. However, the evolutionary distance of CsPAP1 (c124394.graph_c0) was further from the other four tea PAP1 proteins. The qRT-PCR results confirmed that four *CsPAP1* genes (c112219.graph_c0, c53572.graph_c0, c121522.graph_c0, and c124394.graph_c0) were significantly up-regulated in TGY (Wei) and TGY (Wang) compared with BS. The products of these genes may contribute to anthocyanin accumulation. The PlantTFDB [26] BLAST results indicated that three CsPAP1s (encoded by c112219.graph_c0, c53572.graph_c0, and c121522.graph_c0) may coordinately regulate the biosynthesis of phenylpropanoid-derived compounds such as anthocyanin and catechins via an interaction with *EGL3* in TGY. Thus, we speculated that anthocyanin biosynthesis in TGY is concurrently regulated by PAP1 TFs, and that the expression level of *CsPAP1* is positively correlated with catechins production. This would explain the difference in leaf color and flavor between the two original TGY samples and BS. Further research is required to identify the mechanisms that control the interactions responsible for producing specialized metabolites in these metabolic pathways.

## Conclusions

Transcriptome analyses and HPLC and GC-MS analyses of TGY (Wei), TGY (Wang), and BS revealed various pigments (chlorophylls, carotenoids, and anthocyanin), metabolites (catechins, caffeine, and limonene), and related metabolic pathways (flavonoids metabolism, caffeine metabolism, and limonene degradation) responsible for the leaf color and secondary metabolite profiles of TGY and BS. Our results showed that, compared with BS, TGY contains higher levels of anthocyanin, carotenoids, catechins, caffeine, and limonene. High contents of anthocyanin and carotenoids contribute to the deeper purple leaf color of TGY than BS. We also analyzed the expression profiles genes involved in flavonoid metabolism, caffeine metabolism, and limonene degradation pathways that were differentially expressed among the three tea plants. The results suggested that the expression of DEGs involved in the flavonoid, caffeine, and limonene metabolic pathways might be correlated with the accumulation of related metabolites in TGY. Further analyses of TFs revealed that the CsPAP1 TF may participate in regulating the flavonoid and anthocyanin metabolic pathways. High transcript levels of *CsPAP1* corresponded to increased accumulation of anthocyanin and catechins in TGY. Therefore, the sensory quality of TGY (Wei) and TGY (Wang) might be better than that of BS because of the accumulation of flavonoids, caffeine, and limonene metabolites. These metabolites did not show significantly different concentrations between TGY (Wei) and TGY (Wang), suggesting that these two plants may be sister strains of ‘Tieguanyin’. Our study underpins future research on the relationships and molecular mechanisms that contribute to the accumulation of secondary metabolites in tea. These findings also highlight that the expression levels of genes encoding important enzymes in secondary metabolic pathways affect the formation of the “Yin Rhyme” flavor in ‘Tieguanyin’.

## Additional files

Additional file 1: **Figure S1.** Phenotype of original tea plants. (A): “Wei shuo” ‘Tieguanyin’ (*Camellia sinensis* cv. ‘Tieguanyin’); (B) “Wang shuo” ‘Tieguanyin’ (*C. sinensis* cv. ‘Tieguanyin’); (C) ‘Benshan’ (*C. sinensis* cv. ‘Benshan’). (TIF 8541 kb)

Additional file 2: **Table S1.** Primers used for qRT-PCR analyses. (DOC 66 kb)

Additional file 3: **Table S2.** Main biological characteristics of TGY (Wei), TGY (Wang), and BS. (DOC 37 kb)

Additional file 4: **Table S3.** Read count data in transcriptome dataset. (XLSX 4399 kb)

Additional file 5: **Figure S2.** Gene ontology (GO) analysis of all unigenes. (PNG 490 kb)

Additional file 6: **Figure S3.** Cluster of orthologous groups (COG) analysis of all unigenes. (TIF 1749 kb)

Additional file 7: **Figure S4**. Number of transcription factors (TFs) identified in TGY (Wei), TGY (Wang), and BS. (TIF 1075 kb)

Additional file 8: **Figure S5.** Hierarchical clustering analysis of relative expression levels of differentially expressed genes (DEGs) in TGY (Wei), TGY (Wang), and BS. (TIF 1323 kb)

Additional file 9: **Table S4.** Differentially expressed genes (DEGs) related to limonene metabolic pathways in TGY (Wei), TGY (Wang), and BS. (DOC 16 kb)

## Abbreviations

DEGs: Differentially expressed genes
FPKM: Fragments per kilobase of exon per million fragments
GC-MS: Gas chromatography-mass spectrometry
HMM: Hidden Markov model
HPLC: High performance liquid chromatography
mRNA: messenger RNA
qRT-PCR: Quantitative real-time polymerase chain reaction

## References

[1] Wang L, Yue C, Cao HL, Zhou YH, Zeng JM, Yang YJ, Wang XC (2014). Biochemical and transcriptome analyses of a novel chlorophyll-deficient chlorina tea plant cultivar. BMC Plant Biol.

[2] Wei CL, Yang H, Wang SB, Zhao J, Liu C, Gao LP, Xia EH, Lu Y, Tai YL, She GB (2018). Draft genome sequence of *Camellia sinensis* var. *sinensis* provides insights into the evolution of the tea genome and tea quality. Proc Natl Acad Sci U S A.

[3] Huang JA, Shi ZP, Shi Y, Gu JP, Chen JH, Gong YS (2003). Study on sense experience and chemical characteristics of Yanyun and Yinyun in oolong tea. J Hunan Agric Univ (Nat Sci).

[4] Xin W, Hong YC, Lu L, Wang FQ (2012). Sensory evaluation and chemical components analysis on Tieguanyin of different shaking styles in Anxi for Yinyun. J Wuyi Univ.

[5] Liu SR, Liu HW, Wu AL, Hou Y, An YL, Wei CL (2017). Construction of fingerprinting for tea plant (*Camellia sinensis*) accessions using new genomic SSR markers. Mol Breeding.

[6] Liu SR, An YL, Li FD, Li SJ, Liu LL, Zhou QY, Zhao SQ, Wei CL (2018). Genome-wide identification of simple sequence repeats and development of polymorphic SSR markers for genetic studies in tea plant (*Camellia sinensis*). Mol Breeding..

[7] Vaidya K, Ghosh A, Kumar V, Chaudhary S, Srivastava N, Katudia K, Tiwari T, Chikara SK (2012). *De novo* transcriptome sequencing in *Trigonella foenum*-*graecum* L. to identify genes involved in the biosynthesis of diosgenin. Plant Genome.

[8] Liu MY, Qiao GR, Jiang J, Yang HQ, Xie LH, Xie JZ, Zhuo RY (2012). Transcriptome sequencing and d*e novo* analysis for ma bamboo (*Dendrocalamus latiflorus* Munro) using the Illumina platform. PLoS One.

[9] Harismendy O, Ng PC, Strausberg RL, Wang X, Stockwell TB, Beeson KY, Schork NJ, Murray SS, Topol EJ, Levy S (2009). Evaluation of next generation sequencing platforms for population targeted sequencing studies. Genome Biol.

[10] Hendre PS, Kamalakannan R, Varghese M (2012). High-throughput and parallel SNP discovery in selected candidate genes in Eucalyptus camaldulensis using Illumina NGS platform. Plant Biotechnol J.

[11] Shi CY, Yang H, Wei CL, Yu O, Zhang ZZ, Jiang CJ, Sun J, Li YY, Chen Q, Xia T (2011). Deep sequencing of the *Camellia sinensis* transcriptome revealed candidate genes for major metabolic pathways of tea-specific compounds. BMC Genomics.

[12] Wu ZJ, Li XH, Liu ZW, Xu ZS, Zhuang J. *De novo* assembly and transcriptome characterization: novel insights into catechins biosynthesis in *Camellia sinensis*. BMC Plant Biol. 2014;14:277. 10.1186/s12870-014-0277-4 PMC4203915 25316555

[13] Wang L, Cao HL, Chen CS, Yue C, Hao XY, Yang YJ, Wang XC (2016). Complementary transcriptomic and proteomic analyses of a chlorophyll-deficient tea plant cultivar reveal multiple metabolic pathway changes. J Proteome.

[14] Deng XJ, Zhang HQ, Wang Y, He F, Liu JL, Xiao X, Shu ZF, Li W, Wang GH, Wang GL (2014). Mapped clone and functional analysis of leaf-color gene *Ygl7* in a rice hybrid (*Oryza sativa* L. ssp. *indica*). PLoS One.

[15] Zhou QQ, Sun WJ, Lai ZX (2016). Differential expression of genes in purple-shoot tea tender leaves and mature leaves during leaf growth. J Sci Food Agr.

[16] Tai YL, Wei CL, Yang H, Zhang L, Chen Q, Deng WW, Wei S, Zhang J, Fang CB, Ho CT (2015). Transcriptomic and phytochemical analysis of the biosynthesis of characteristic constituents in tea (*Camellia sinensis*) compared with oil tea (*Camellia oleifera*). BMC Plant Biol.

[17] Zhu XL, Chen B, Luo XB, Yao SZ (2003). Determination of theanine in tea by reversed-phase high performance liquid chromatography. Chin J Chrom.

[18] Hu CJ, Li D, Ma YX, Zhang W, Lin C, Zheng XQ, Liang YR, Lu JL (2018). Formation mechanism of the oolong tea characteristic aroma during bruising and withering treatment. Food Chem.

[19] Lai ZX, Lin YL (2013). Analysis of the global transcriptome of longan (*Dimocarpus longan* Lour.) embryogenic callus using Illumina paired-end sequencing. BMC Genomics.

[20] Grabherr MG, Haas BJ, Yassour M, Levin JZ, Thompson DA, Amit I, Adiconis X, Fan L, Raychowdhury R, Zeng Q (2011). Full-length transcriptome assembly from RNA-Seq data without a reference genome. Nat Biotechnol.

[21] Kim D, Langmead B, Salzberg SL (2015). HISAT: a fast spliced aligner with low memory requirements. Nat Methods.

[22] Xia EH, Zhang HB, Sheng J, Li K, Zhang QJ, Kim C, Zhang Y, Liu Y, Zhu T, Li W (2017). The tea tree genome provides insights into tea flavor and independent evolution of caffeine biosynthesis. Mol Plant.

[23] Xie C, Mao XZ, Huang JJ, Ding Y, Wu JM, Dong S, Kong L, Gao G, Li CY, Wei LP (2011). KOBAS 2.0: a web server for annotation and identification of enriched pathways and diseases. Nucleic Acids Res.

[24] Rice P, Longden I, Bleasby A (2000). EMBOSS: the european molecular biology open software suite. Trends Genet.

[25] Mistry J, Finn RD, Eddy SR, Bateman A, Punta M (2013). Challenges in homology search: HMMER3 and convergent evolution of coiled-coil regions. Nucleic Acids Res.

[26] Jin JP, Tian F, Yang DC, Meng YQ, Kong L, Luo JC, Gao G (2017). PlantTFDB 4.0: toward a central hub for transcription factors and regulatory interactions in plants. Nucleic Acids Res.

[27] Langmead B, Salzberg SL (2011). Fast gapped-read alignment with bowtie 2. Nat Methods.

[28] Li B, Dewey CN (2011). RSEM: accurate transcript quantification from RNA-Seq data with or without a reference genome. BMC Bioinformatics.

[29] Leng N, Dawson JA, Thomson JA, Ruotti V, Rissman AI, Smits BM, Haag JD, Gould MN, Stewart RM, Kendziorski C (2013). EBSeq: an empirical Bayes hierarchical model for inference in RNA-seq experiments. Bioinformatics..

[30] Storey JD (2003). The positive false discovery rate: a bayesian interpretation and the q-value. Ann Stat.

[31] Altschul SF, Madden TL, Schäffer AA, Zhang J, Zhang Z, Miller W, Lipman DJ (1997). Gapped BLAST and PSI-BLAST: a new generation of protein database search programs. Nucleic Acids Res.

[32] Kumar S, Stecher G, Tamura K (2016). MEGA7: molecular evolutionary genetics analysis version 7.0 for bigger datasets. Mol Biol Evol.

[33] Lin YL, Lai ZX (2010). Reference gene selection for qPCR analysis during somatic embryogenesis in longan tree. Plant Sci.

[34] Zheng XQ, Li QS, Xiang LP, Liang YR (2016). Recent advances in volatiles of teas. Molecules..

[35] Yang ZY, Baldermann S, Watanabe N (2013). Recent studies of the volatile compounds in tea. Food Res Int.

[36] Xu QS, He YX, Yan XM, Zhao SQ, Zhu JY, Wei CL (2018). Unraveling a crosstalk regulatory network of temporal aroma accumulation in tea plant (*Camellia sinensis*) leaves by integration of metabolomics and transcriptomics. Environ Exp Bot.

[37] Shin DH, Choi M, Kim K, Bang G, Cho M, Choi SB, Choi G, Park YI (2013). HY5 regulates anthocyanin biosynthesis by inducing the transcriptional activation of the MYB75/PAP1 transcription factor in *Arabidopsis*. FEBS Lett.

[38] Zvi MM, Shklarman E, Masci T, Kalev H, Debener T, Shafir S, Ovadis M, Vainstein A (2012). PAP1 transcription factor enhances production of phenylpropanoid and terpenoid scent compounds in rose flowers. New Phytol.

[39] Lee DW, O’Keefe J, Holbrook NM, Feild TS (2003). Pigment dynamics and autumn leaf senescence in a New England deciduous forest, eastern USA. Ecol Res.

[40] Lightbourn GJ, Griesbach RJ, Novotny JA, Clevidence BA, Rao DD, Stommel JR (2008). Effects of anthocyanin and carotenoid combinations on foliage and immature fruit color of *Capsicum annuum* L. J Hered.

[41] Lu YF, Zhang ML, Meng XN, Wan HH, Zhang J, Tian J, Hao SX, Jin KN, Yao YC (2015). Photoperiod and shading regulate coloration and anthocyanin accumulation in the leaves of *malus* crabapples. Plant Cell Tiss Org.

[42] Li CF, Xu YX, Ma JQ, Jin JQ, Huang DJ, Yao MZ, Ma CL, Chen L (2016). Biochemical and transcriptomic analyses reveal different metabolite biosynthesis profiles among three color and developmental stages in 'Anji Baicha' (*Camellia sinensis*). BMC Plant Biol.

[43] Iwashina T (2015). Contribution to flower colors of flavonoids including anthocyanins: a review. Nat Prod Commun.

[44] Winkel-Shirley B (2001). Flavonoid biosynthesis. A colorful model for genetics, biochemistry, cell biology, and biotechnology. Plant Physiol.

[45] Karageorgou P, Manetas Y (2006). The importance of being red when young: anthocyanins and the protection of young leaves of *Quercus coccifera* from insect herbivory and excess light. Tree Physiol.

[46] Jun SY, Sattler SA, Cortez GS, Vermerris W, Sattler SE, Kang C (2018). Biochemical and structural analysis of substrate specificity of a phenylalanine ammonia-lyase. Plant Physiol.

[47] Ferrer JL, Austin MB, Stewart C, Noel JP (2008). Structure and function of enzymes involved in the biosynthesis of phenylpropanoids. Plant Physiol Bioch..

[48] Anson JG, Gilbert HJ, Oram JD, Minton NP (1987). Complete nucleotide sequence of the Rhodosporidium toruloides gene coding for *phenylalanine ammonia-lyase*. Gene..

[49] Song J, Wang ZZ (2009). Molecular cloning, expression and characterization of a *phenylalanine ammonia-lyase* gene (*SmPAL1*) from *Salvia miltiorrhiza*. Mol Biol Rep.

[50] Dong CJ, Cao N, Shang QM, Zhang ZG (2016). *Phenylalanine ammonia-lyase* gene families in cucurbit species: structure, evolution, and expression. J Integr Agr.

[51] Vogt T (2010). Phenylpropanoid biosynthesis. Mol Plant.

[52] Bell-Lelong DA, Cusumano JC, Meyer K, Chapple C (1997). *Cinnamate-4-hydroxylase* expression in *Arabidopsis* (regulation in response to development and the environment). Plant Physiol.

[53] Mizutani M, Ohta D, Sato R (1997). Isolation of a cDNA and a genomic clone encoding cinnamate 4-hydroxylase from *Arabidopsis* and its expression manner in planta. Plant Physiol.

[54] Liu M, Tian HL, Wu JH, Cang RR, Wang RX, Qi XH, Xu Q, Chen XH (2015). Relationship between gene expression and the accumulation of catechin during spring and autumn in tea plants (*Camellia sinensis* L.). Hortic Res.

[55] De Lorenzis G, Rustioni L, Parisi SG, Zoli F, Brancadoro L (2016). Anthocyanin biosynthesis during berry development in corvina grape. Sci Hortic.

[56] Zhou TS, Zhou R, Yu YB, Xiao Y, Li DH, Xiao B, Yu O, Yang YJ (2016). Cloning and characterization of a *flavonoid 3′-hydroxylase* gene from tea plant (*Camellia sinensis*). Int J Mol Sci.

[57] Han YH, Huang KY, Liu YJ, Jiao TM, Ma GL, Qian YM, Wang PQ, Dai XL, Gao LP, Xia T (2017). Functional analysis of two *flavanone-3-hydroxylase* genes from *Camellia sinensis*: a critical role in flavonoid accumulation. Genes..

[58] Moriguchi T, Kita M, Tomono Y, Endo-Inagaki T, Omura M. Gene expression in flavonoid biosynthesis: correlation with flavonoid accumulation in developing citrus fruit. Physiol Plantarum. 2001;111(1):66–74.

[59] Pelt JL, Downes WA, Schoborg RV, McIntosh CA (2003). Flavanone 3-hydroxylase expression in *Citrus paradisi* and *Petunia hybrida* seedlings. Phytochemistry..

[60] Pelletier MK, Shirley BW. Analysis of flavanone 3-hydroxylase in *Arabidopsis* seedlings (Coordinate regulation with chalcone synthase and chalcone isomerase). Plant Physiol. 1996;111(1):339–345. 10.1104/pp.111.1.339 PMC157841 8685272

[61] Rothenberg DO, Yang HJ, Chen MB, Zhang WT, Zhang LY. Metabolome and transcriptome sequencing analysis reveals anthocyanin metabolism in pink flowers of anthocyanin-rich Tea (*Camellia sinensis*). Molecules. 2019;24(6):1064. 10.3390/molecules24061064 PMC6471635 30889908

[62] Sun BM, Zhu ZS, Cao PR, Hao C, Chen CM, Xin Z, Mao YH, Lei JJ, Jiang YP, Wei M (2016). Purple foliage coloration in tea (*Camellia sinensis* L.) arises from activation of the R2R3-MYB transcription factor CsAN1. Sci Rep.

[63] Kumar V, Nadda G, Kumar S, Yadav SK (2013). Transgenic tobacco overexpressing tea cDNA encoding dihydroflavonol 4-reductase and anthocyanidin reductase induces early flowering and provides biotic stress tolerance. PLoS One.

[64] Guo YQ, Chang XJ, Zhu C, Zhang ST, Li XZ, Fu HF, Chen CS, Lin YL, Lai ZX. *De novo* transcriptome combined with spectrophotometry and gas chromatography-mass spectrometer (GC-MS) reveals differentially expressed genes during accumulation of secondary metabolites in purple-leaf tea (*Camellia sinensis* cv Hongyafoshou). J Hortic Sci Biotechnol. 2019;94(3):349–67.

[65] Punyasiri PA, Abeysinghe IS, Kumar V, Treutter D, Duy D, Gosch C, Martens S, Forkmann G, Fischer TC (2004). Flavonoid biosynthesis in the tea plant *Camellia sinensis*: properties of enzymes of the prominent epicatechin and catechin pathways. Arch Biochem Biophys.

[66] Chao N, Li N, Qi Q, Li S, Lv T, Jiang XN, Gai Y (2017). Characterization of the *cinnamoyl-CoA reductase* (*CCR*) gene family in *Populus tomentosa* reveals the enzymatic active sites and evolution of CCR. Planta..

[67] Ponniah SK, Shang Z, Akbudak MA, Srivastava V, Manoharan M (2017). Down-regulation of hydroxycinnamoyl CoA: shikimate hydroxycinnamoyl transferase, cinnamoyl CoA reductase, and cinnamyl alcohol dehydrogenase leads to lignin reduction in rice (*Oryza sativa* L. ssp. *japonica* cv. Nipponbare). Plant Biotechnol Rep.

[68] Kallscheuer N, Vogt M, Bott M, Marienhagen J (2017). Functional expression of plant-derived O-methyltransferase, flavanone 3-hydroxylase, and flavonol synthase in *Corynebacterium glutamicum* for production of pterostilbene, kaempferol, and quercetin. J Biotechnol.

[69] Zhang CY, Liu HC, Jia CG, Liu YJ, Wang FT, Wang JY (2016). Cloning, characterization and functional analysis of a flavonol synthase from *Vaccinium corymbosum*. Trees..

[70] Gagné S, Lacampagne S, Claisse O, Gény L (2009). *Leucoanthocyanidin reductase* and *anthocyanidin reductase* gene expression and activity in flowers, young berries and skins of *Vitis vinifera* L. cv. Cabernet-sauvignon during development. Plant Physiol Bioch..

[71] Maugé C, Granier T, D'Estaintot BL, Gargouri M, Manigand C, Schmitter J, Chaudière J, Gallois B (2010). Crystal structure and catalytic mechanism of leucoanthocyanidin reductase from Vitis vinifera. J Mol Biol.

[72] Obanda M, Owuor PO, Taylor SJ (1997). Flavanol composition and caffeine content of green leaf as quality potential indicators of Kenyan black teas. J Sci Food Agr..

[73] Suteerapataranon S, Butsoongnern J, Punturat P, Jorpalit W, Thanomsilp C (2009). Caffeine in Chiang Rai tea infusions: effects of tea variety, type, leaf form, and infusion conditions. Food Chem.

[74] Fujimori N, Ashihara H. Biosynthesis of caffeine in flower buds of *Camellia sinensis*. Ann Bot. 1993;71(3):279–84.

[75] Mohanpuria P, Kumar V, Yadav SK (2010). Tea caffeine: metabolism, functions, and reduction strategies. Food Sci Biotechnol.

[76] Mohanpuria P, Kumar V, Joshi R, Gulati A, Ahuja PS, Yadav SK (2009). Caffeine biosynthesis and degradation in tea [*Camellia sinensis* (L.) O. Kuntze] is under developmental and seasonal regulation. Mol Biotechnol.

[77] Suzuki T, Ashihara H, Waller GR (1992). Purine and purine alkaloid metabolism in *Camellia* and *Coffea* plants. Phytochemistry..

[78] Li CF, Zhu Y, Yu Y, Zhao QY, Wang SJ, Wang XC, Yao MZ, Luo D, Li X, Chen L (2015). Global transcriptome and gene regulation network for secondary metabolite biosynthesis of tea plant (*Camellia sinensis*). BMC Genomics.

[79] Nishimura K, Ashihara H (1993). IMP dehydrogenase from tea leaves and suspension-cultured *Catharanthus roseus* cells. Phytochemistry..

[80] Keya CA, Crozier A, Ashihara H (2003). Inhibition of caffeine biosynthesis in tea (*Camellia sinensis*) and coffee (*Coffea arabica*) plants by ribavirin. FEBS Lett.

[81] Zrenner R, Stitt M, Sonnewald U, Boldt R (2006). Pyrimidine and purine biosynthesis and degradation in plants. Annu Rev Plant Biol.

[82] Koyama Y, Tomoda Y, Kato M, Ashihara H (2003). Metabolism of purine bases, nucleosides and alkaloids in theobromine-forming Theobroma cacao leaves. Plant Physiol Bioch.

[83] Watanabe S, Kounosu Y, Shimada H, Sakamoto A (2014). Arabidopsis *xanthine dehydrogenase* mutants defective in purine degradation show a compromised protective response to drought and oxidative stress. Plant Biotechnol.

[84] Watanabe S, Nakagawa A, Izumi S, Shimada H, Sakamoto A (2010). RNA interference-mediated suppression of xanthine dehydrogenase reveals the role of purine metabolism in drought tolerance in *Arabidopsis*. FEBS Lett.

[85] Lücker J, El Tamer MK, Schwab W, Verstappen FWA, Van der Plas LHW, Bouwmeester HJ, Verhoeven HA (2002). Monoterpene biosynthesis in lemon (*Citrus limon*). cDNA isolation and functional analysis of four monoterpene synthases. Eur J Biochem.

[86] Kjonaas R, Croteau R (1983). Demonstration that limonene is the first cyclic intermediate in the biosynthesis of oxygenated p-menthane monoterpenes in *Mentha piperita* and other *Mentha* species. Arch Biochem Biophys.

[87] Fischbach RJ, Zimmer I, Steinbrecher R, Pfichner A, Schnitzler JP (2000). Monoterpene synthase activities in leaves of *Picea abies* (L.) karst. And *Quercus ilex* L. Phytochemistry..

[88] Duetz WA, Bouwmeester H, van Beilen JB, Witholt B (2003). Biotransformation of limonene by bacteria, fungi, yeasts, and plants. Appl Microbiol Biot.

[89] Chang HC, Gage DA, Oriel PJ (1995). Cloning and expression of a limonene degradation pathway from *Bacillus stearothermophilus* in *Escherichia coli*. J Food Sci.

[90] van der Werf MJ, Swarts HJ, de Bont JA (1999). Rhodococcus erythropolis DCL14 contains a novel degradation pathway for limonene. Appl Environ Microb.

[91] Xie J, Deng LL, Zhou YH, Yao SX, Zeng KF (2018). Analysis of changes in volatile constituents and expression of genes involved in terpenoid metabolism in oleocellosis peel. Food Chem.

[92] Xu WJ, Dubos C, Lepiniec L (2015). Transcriptional control of flavonoid biosynthesis by MYB-bHLH-WDR complexes. Trends Plant Sci.

[93] Gonzalez A, Zhao M, Leavitt JM, Lloyd AM (2008). Regulation of the anthocyanin biosynthetic pathway by the TTG1/bHLH/Myb transcriptional complex in *Arabidopsis* seedlings. Plant J.

[94] Yao GF, Ming ML, Allan AC, Gu C, Li LT, Wu X, Wang RZ, Chang YJ, Qi KJ, Zhang SL (2017). Map-based cloning of the pear gene *MYB114* identifies an interaction with other transcription factors to coordinately regulate fruit anthocyanin biosynthesis. Plant J.

