# Supplementary figures and images for: De novo transcriptome and phytochemical analyses reveal differentially expressed genes and characteristic secondary metabolites in the original oolong tea (Camellia sinensis) cultivar ‘Tieguanyin’ compared with cultivar ‘Benshan’
Source: BMC Genomics. 2019 Apr 3;20:265. doi: 10.1186/s12864-019-5643-z (PMC6446291; doi:10.1186/s12864-019-5643-z)

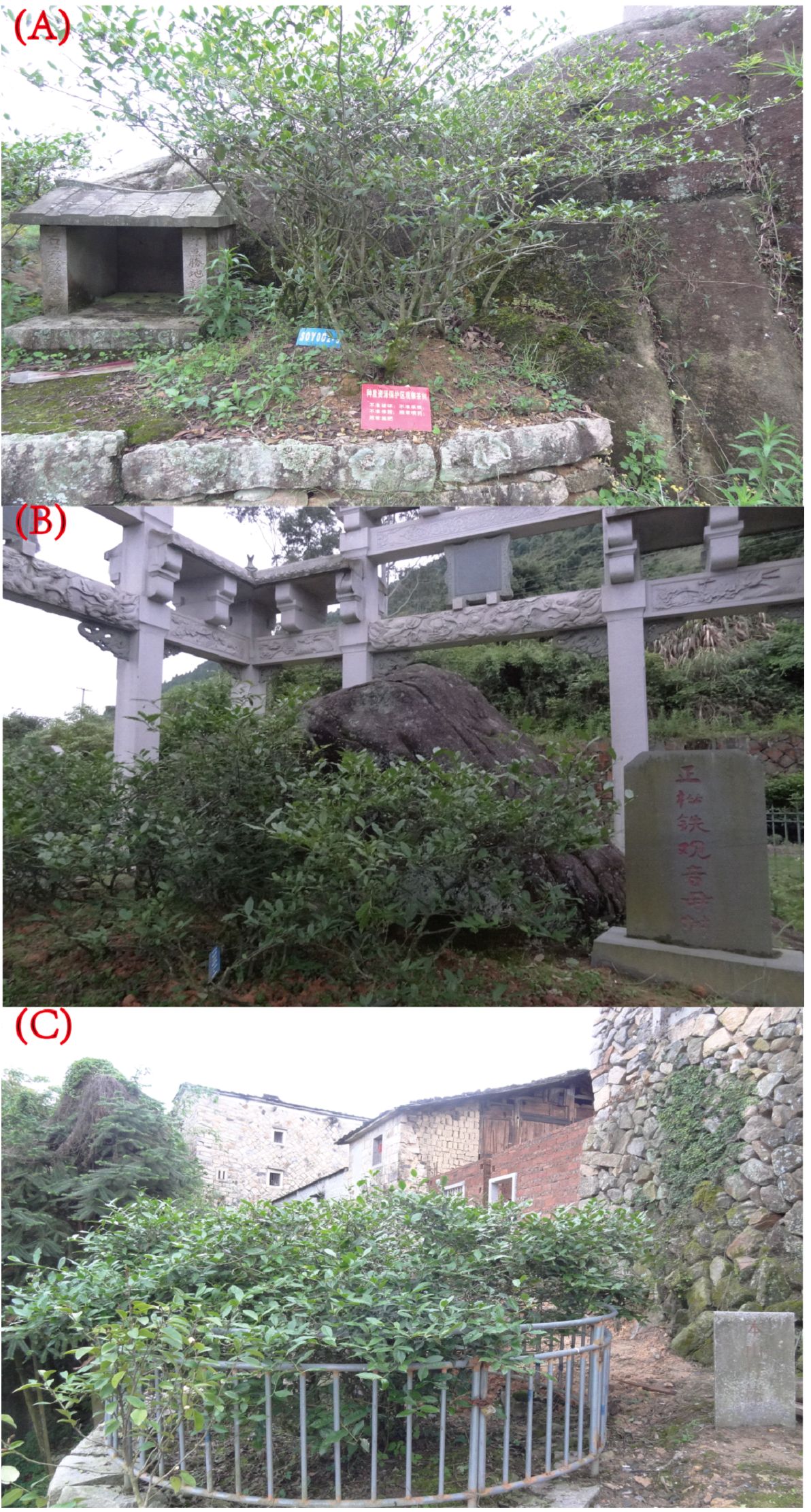

Supplement: Supplementary file 1 — Figure S1. Phenotype of original tea plants. (A): “Wei shuo” ‘Tieguanyin’ (Camellia sinensis cv. ‘Tieguanyin’); (B) “Wang shuo” ‘Tieguanyin’ (C. sinensis cv. ‘Tieguanyin’); (C) ‘Benshan’ (C. sinensis cv. ‘Benshan’). (TIF 8541 kb) [file 12864_2019_5643_MOESM1_ESM.tif]

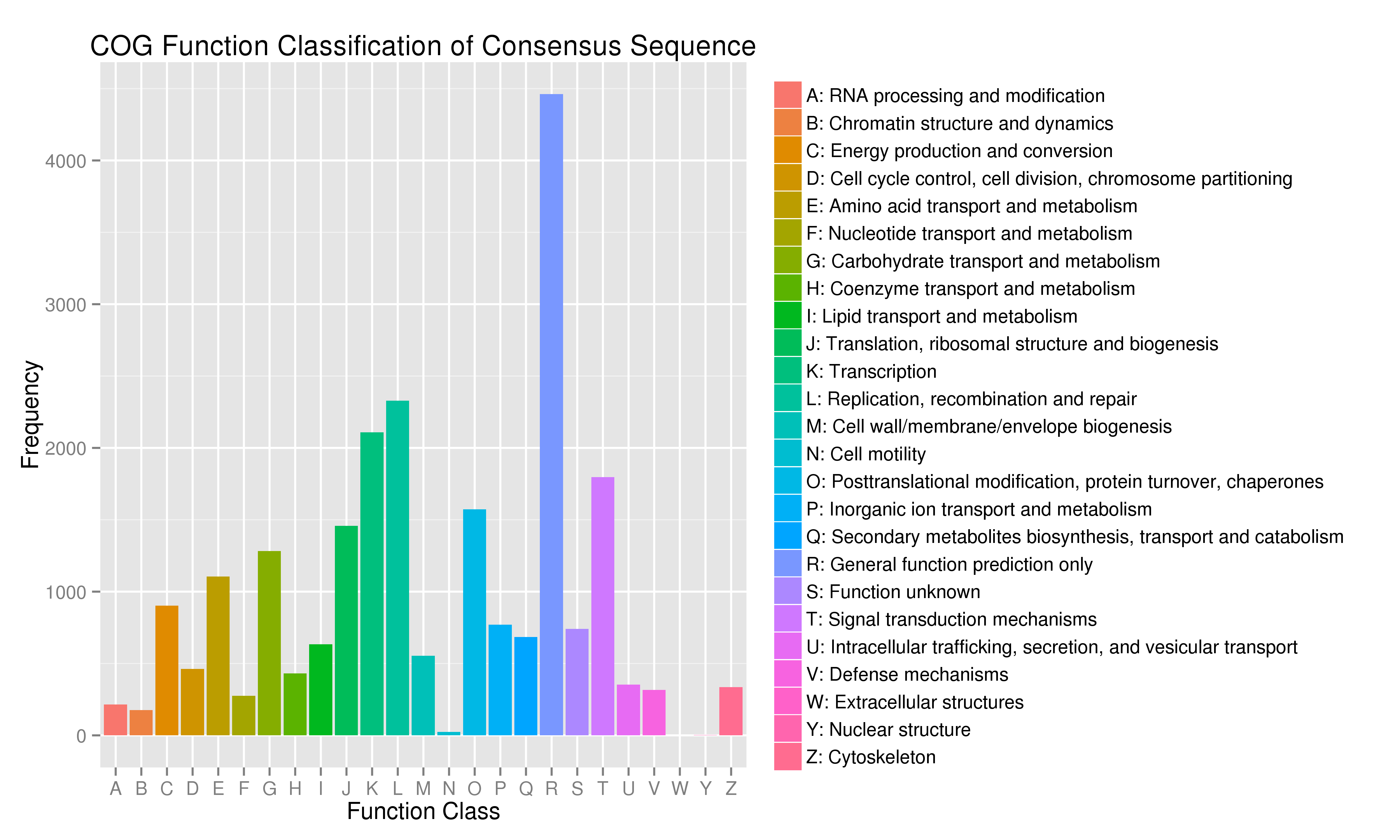

Supplement: Supplementary file 5 — Figure S2. Gene ontology (GO) analysis of all unigenes. (PNG 490 kb) [file 12864_2019_5643_MOESM5_ESM.png]

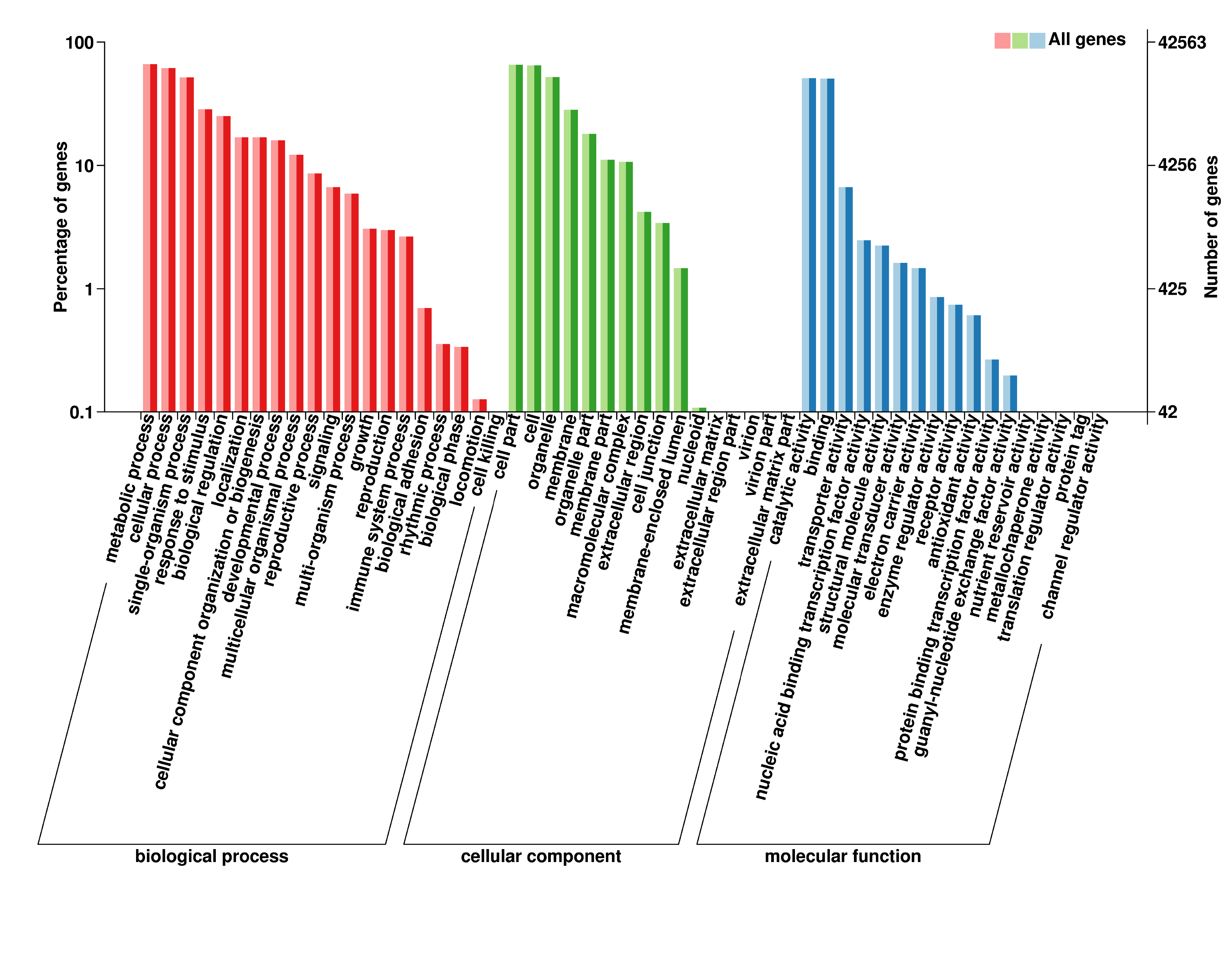

Supplement: Supplementary file 6 — Figure S3. Cluster of orthologous groups (COG) analysis of all unigenes. (TIF 1749 kb) [file 12864_2019_5643_MOESM6_ESM.tif]

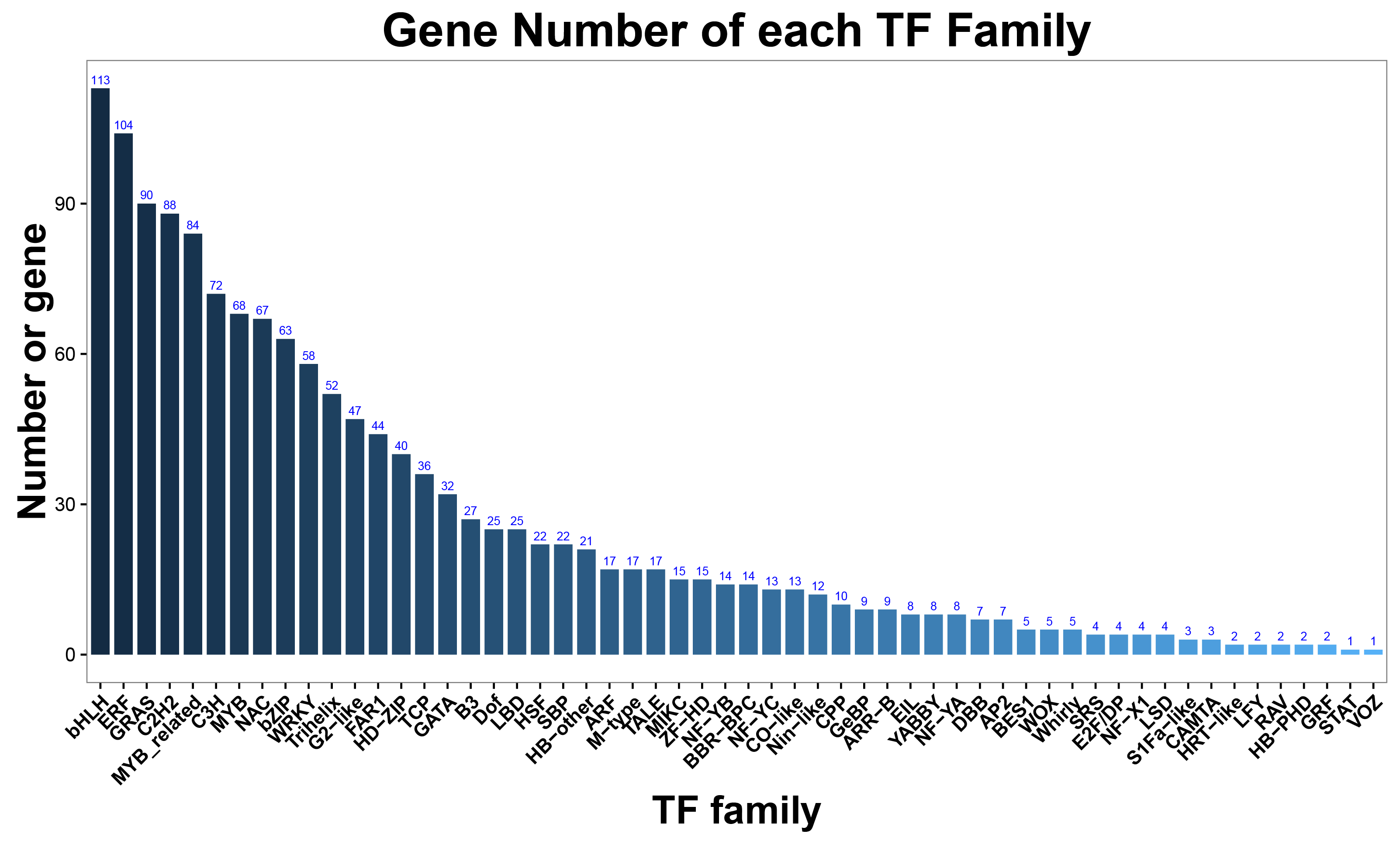

Supplement: Supplementary file 7 — Figure S4. Number of transcription factors (TFs) identified in TGY (Wei), TGY (Wang), and BS. (TIF 1075 kb) [file 12864_2019_5643_MOESM7_ESM.tif]

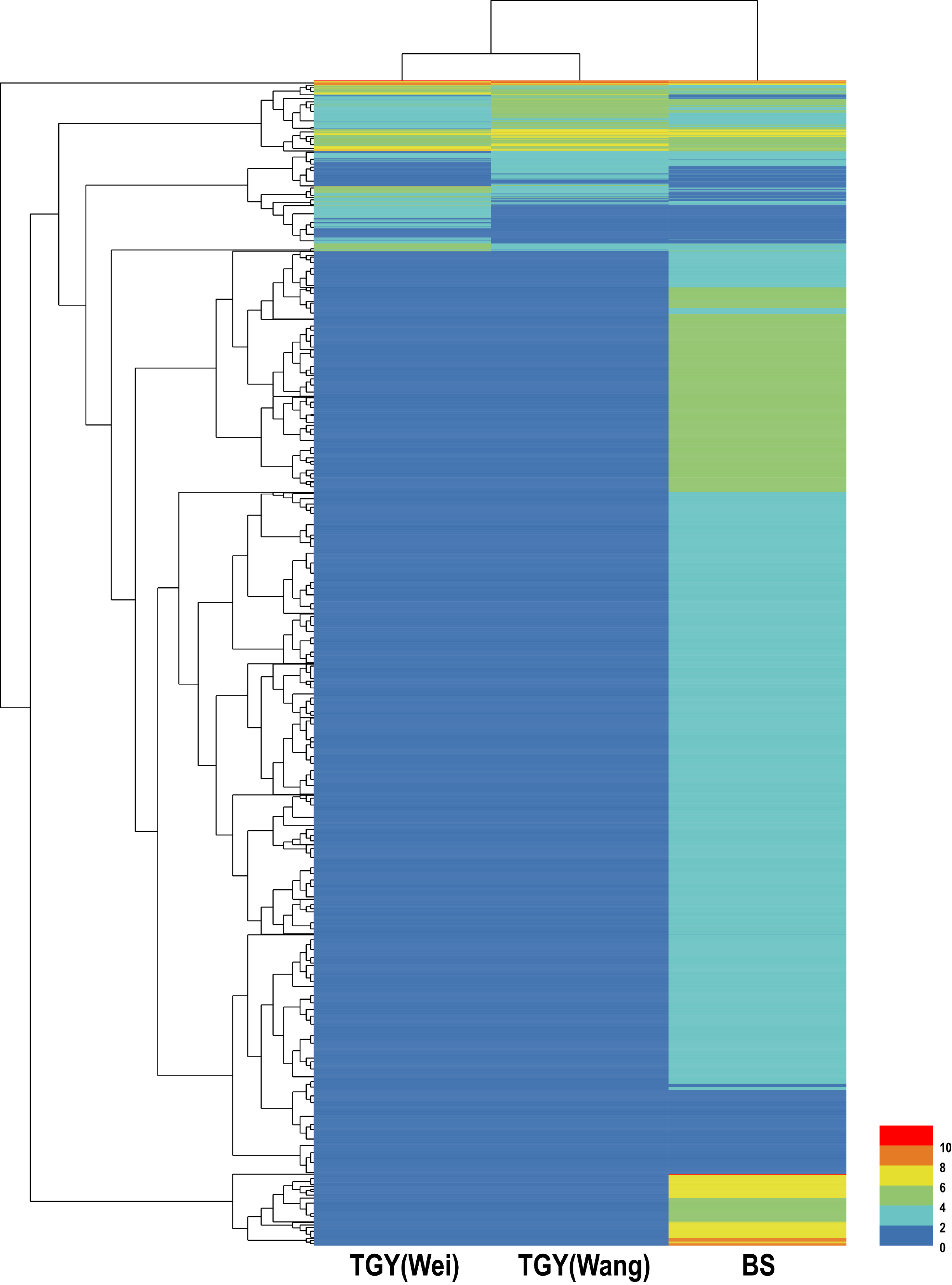

Supplement: Supplementary file 8 — Figure S5. Hierarchical clustering analysis of relative expression levels of differentially expressed genes (DEGs) in TGY (Wei), TGY (Wang), and BS. (TIF 1323 kb) [file 12864_2019_5643_MOESM8_ESM.tif]
