# Supplementary material for: De novo transcriptome and phytochemical analyses reveal differentially expressed genes and characteristic secondary metabolites in the original oolong tea (Camellia sinensis) cultivar ‘Tieguanyin’ compared with cultivar ‘Benshan’
Source: BMC Genomics. 2019 Apr 3;20:265. doi: 10.1186/s12864-019-5643-z (PMC6446291; doi:10.1186/s12864-019-5643-z)
Supplement: Supplementary file 2 — Table S1. Primers used for qRT-PCR analyses. (DOC 66 kb) [file 12864_2019_5643_MOESM2_ESM.doc]

Additional file 2: Table S1. Primers used for qRT-PCR analyses.

| **Classification** | **Gene** | **Gene ID** | **Forward primer (5'-3')** | **Reverse primer (5'-3')** |
| --- | --- | --- | --- | --- |
| Flavonoid metabolism | *PAL* | c83716.graph_c0 | ACTCTCCAAAGCACTTCTCGT | CGAGTTCTCGTTGATCATGC |
|  | *C4H* | c111306.graph_c0 | GCACGATCCCATTGGTCTTC | GGTGAGCACTGGAGGAAGAT |
|  | *4CL* | c116268.graph_c0 | GCCGACGAAGAGCTTTTCAT | ACAAATGCCACCGGAACTTC |
|  | *CHS* | c116615.graph_c1 | CGTCCAAGATGAAGAGCACG | AAGAACATCGAGAAGGCCCT |
|  | *F3H* | c107454.graph_c0 | AGAATGTCTCAGCTGCCACT | AATCCTCCAAGAGTGACGGG |
|  | *FLS* | c114631.graph_c0 | TGATTGTTTCGTGGAGTGCC | TGCTTGCCATGTCCACTAG |
|  | *DFR* | c122980.graph_c0 | TACCCCATTTACAGCAGGC | TACACAGTAAACGCCACCCT |
|  | *F3'H* | c50625.graph_c0 | ACCAGATGCTTTCCTCGTG | CAGCGTCAAATCAGGTCGAG |
|  | *ANS* | c97715.graph_c0 | ATTTCCCTCCAAGCACCTG | TTTTCTGCGAACCACCCAAG |
|  | *ANR* | c95232.graph_c0 | GAAGCCACTCCATTGCCTTT | CTGTGTAGCCCTTTTCCAGC |
|  | *CCR* | c86572.graph_c0 | TTGAGGATGTGGAGTGTGCT | TGTTGATGAGAGCTGCTGG |
| Caffeine metabolism | *AMPD* | c106010.graph_c0 | CAGATCCAGTGGCTGCAAAT | TTGGCTTAGGAGTGCAAGG |
|  | *IMPDH* | c108398.graph_c0 | ACTCATCCCACCAACCACTT | ACAATCGTCGGAGTCCTGTT |
|  | *IMPDH* | c94772.graph_c0 | TACGTGCAGTTCCCTCAGAG | ACCCGTAAAGTGCTTGCCT |
|  | *IMPDH* | c98658.graph_c0 | GTCTTGCAATAGGTCGAGGC | CGCCGGCAACATTCTTCTT |
|  | *IMPDH* | c107967.graph_c1 | CTCCACTCCATCGCATTTCC | AATGGTTGGAGAGGCTAGC |
|  | *SAM* | c107073.graph_c0 | TTCCAGTCCTGTACGTGTCC | CATGGTGGTGGTGCTTTCTC |
|  | *SAM* | c71387.graph_c0 | TCAAGCCCGTGATACCAGAG | CACCTTGGTTGGATCCTTGC |
|  | *5'-Nase* | c108997.graph_c0 | ACCCTGGACTCTGATGTTGG | AGCCCTCGTTACCTGAAAC |
|  | *XDH* | c66037.graph_c0 | CAAAACGCATAGGTGGTGGT | CGTGCCTCTGTCCTGAAATC |
| limonene degradation | *ALDH* | c104957.graph_c0 | GATGAAGGGACAGCTGCATG | AAGTCTCCCTGAGCTGTCTG |
|  | *ALDH* | c93917.graph_c0 | GCTGAGCAGACACCATTGAG | TTGACCCTGTGAAAGCAAGC |
|  | *HIBADH* | c119421.graph_c0 | GCCTCCTCACCATATCCAC | GCTGGAATTGACACTCGCAT |
| PAP1 transcription factor | *CsPAP1* | c65777.graph_c0 | CGGGAAGTCTGCCTGCTATT | TCAGAGCAGGCTTGAACAG |
|  | *CsPAP1* | c112219.graph_c0 | ATCAGCCGGTCAGTAGTAGC | TCCTCATTGCTACCGAGACC |
|  | *CsPAP1* | c53572.graph_c0 | CCGCACCTTCAACTCGTAAC | GGTTTGTGTGGAGGTTGTGG |
|  | *CsPAP1* | c121522.graph_c0 | ACTTGGTGAGCAATGCATCC | TCTGGCATAGCTGATCGGTT |
|  | *CsPAP1* | c124394.graph_c0 | ATCTGTTCGACCCGGTAGTC | AAAGCCATGGTGCAAAGAGG |
| Reference genes | *GAPDH* |  | TTGGCATCGTTGAGGGTCT | CAGTGGGAACACGGAAAGC |
|  | *Actin* |  | GCCATCTTTGATTGGAATGG | GGTGCCACAACCTTGATCTT |
