# Supplementary material for: De novo transcriptome and phytochemical analyses reveal differentially expressed genes and characteristic secondary metabolites in the original oolong tea (Camellia sinensis) cultivar ‘Tieguanyin’ compared with cultivar ‘Benshan’
Source: BMC Genomics. 2019 Apr 3;20:265. doi: 10.1186/s12864-019-5643-z (PMC6446291; doi:10.1186/s12864-019-5643-z)
Supplement: Supplementary file 3 — Table S2. Main biological characteristics of TGY (Wei), TGY (Wang), and BS. (DOC 37 kb) [file 12864_2019_5643_MOESM3_ESM.doc]

| **Project** | **Contents** | **TGY (Wei)** | **TGY (Wang)** | **BS** |
| --- | --- | --- | --- | --- |
| Germinating and picking periods | time point of one bud and one leaf germinating | April 15 | April 13 | April 2 |
|  | time point of one bud and two leaves germinating | April 20 | April 18 | April 5 |
|  | time point of picking | May 8 | May 7 | May 1 |
| Buds and leaves traits | bud color | green with purple | green with purple | light green |
|  | 1^st^ and 2^nd^ leaves color | green with purple | green with purple | light green with slightly purple |
|  | leaf length (cm) | 7.96 | 7.77 | 7.57 |
|  | leaf width (cm) | 3.09 | 2.92 | 3.42 |
|  | leaf length/width ratio | 2.58 | 2.66 | 2.21 |
|  | leaf shape | long oval | long oval | oval |
|  | leaf quality | thick and soft | thick and soft | thick, hard and brittle |
|  | internode length (cm) | 2.74 | 2.82 | 3.14 |
|  | petiole length (cm) | 0.40 | 0.35 | 0.43 |
|  | hundred-bud weight (one bud and three leaves) (g) | 137.8 | 165.0 | 98.2 |

**Additional file 3: Table S2.** **Main biological characteristics of TGY (Wei), TGY (Wang), and BS.**
