# Supplementary material for: De novo transcriptome and phytochemical analyses reveal differentially expressed genes and characteristic secondary metabolites in the original oolong tea (Camellia sinensis) cultivar ‘Tieguanyin’ compared with cultivar ‘Benshan’
Source: BMC Genomics. 2019 Apr 3;20:265. doi: 10.1186/s12864-019-5643-z (PMC6446291; doi:10.1186/s12864-019-5643-z)
Supplement: Supplementary file 9 — Table S4. Differentially expressed genes (DEGs) related to limonene metabolic pathways in TGY (Wei), TGY (Wang), and BS. (DOC 16 kb) [file 12864_2019_5643_MOESM9_ESM.doc]

**Additional file 9: Table S4. Differentially expressed genes (DEGs) related to limonene metabolic pathways in TGY (Wei), TGY (Wang), and BS.**

| Gene ID | KO ID | Annotation |
| --- | --- | --- |
| c104957.graph_c0 | K00128 | Aldehyde dehydrogenase (ALDH) |
| c93917.graph_c0 | K00128 | Aldehyde dehydrogenase (ALDH) |
| c119421.graph_c0 | K00120 | Hydroxyisobutyrate dehydrogenase (HIBADH) |
